# Inverse-designed dielectric cloaks for entanglement generation

**DOI:** 10.1515/nanoph-2022-0231

**Published:** 2022-08-22

**Authors:** Alberto Miguel-Torcal, Jaime Abad-Arredondo, Francisco J. García-Vidal, Antonio I. Fernández-Domínguez

**Affiliations:** Departamento de Física Teórica de la Materia Condensada and Condensed Matter Physics Center (IFIMAC), Universidad Autónoma de Madrid, E-28049 Madrid, Spain; Institute of High Performance Computing, Agency for Science, Technology, and Research (A*STAR), 138632 Connexis, Singapore

**Keywords:** dielectric cloak, entanglement, inverse design, quantum emitter, topology optimization

## Abstract

We investigate the generation of entanglement between two quantum emitters through the inverse-design engineering of their photonic environment. By means of a topology-optimization approach acting at the level of the electromagnetic Dyadic Green’s function, we generate dielectric cloaks operating at different inter-emitter distances and incoherent pumping strengths. We show that the structures obtained maximize the dissipative coupling between the emitters under extremely different Purcell factor conditions, and yield steady-state concurrence values much larger than those attainable in free space. Finally, we benchmark our design strategy by proving that the entanglement enabled by our devices approaches the limit of maximum-entangled-mixed-states.

## 1 Introduction

The advent of quantum technologies relies on the design and implementation of physical platforms able to support quantum states involving a large number of elemental quantum systems (qubits). Lately, the unprecedented control over light at the sub-wavelength scale enabled by nanophotonics has emerged as a promising resource for this purpose [1, 2]. Thus, different quantum functionalities exploiting the efficiency and tunability of photon-assisted interactions in networks of quantum emitters (QEs, such as atoms, molecules, quantum dots or point defects in crystals) have been proposed [3, 4]. In this context, much research attention has focused on entanglement formation in pairs of qubits [5], a paradigmatic building block for any quantum hardware, through their electromagnetic (EM) coupling in different systems: one-dimensional optical fibers [6–8], photonic crystal cavities [9, 10], plasmonic structures [11–13], metamaterials [14, 15] and chiral waveguides [16, 17]. These schemes found novel and feasible solutions to the long-standing problem of bipartite entanglement maximization [18–20] using the material and geometric toolsets accumulated over the years of nanophotonics research.

Concurrently, the development of inverse design (ID) techniques has made a strong impact in nanophotonics research [21–23]. ID algorithms have proven to be very successful at enhancing, refining and optimizing photonic functionalities [24, 25]. Among the different members of the ID family, topology optimization [26] has contributed greatly to enlarge the design space available for nanoscale optics [27]. Different implementations of this technique have revealed unexpected and counterintuitive opportunities in areas as different as optical circuitry [28], second harmonic generation [29], nanoantennas [30] and metasurfaces [31]. Only very recently, ID has been transferred from the classical to the quantum regime, being exploited to tailor nonclassical degrees of freedom of nanophotonic fields. Thus, initial steps have shown the manipulation of the local density of photonic states [32, 33] and the strength of light–matter interactions [34–36], as well as the implementation of single photon extractors [37] and the suppression of inhomogeneous broadening effects in single-photon transducers [38].

In this article, we apply ID ideas to achieve photon-assisted entanglement generation in QE pairs. In particular, we develop a topology-optimization strategy to obtain dielectric cloaks for the QEs that maximize the Wootters concurrence [5] at different inter-emitter distances. Both QEs are incoherently pumped [39], a technologically relevant configuration [40, 41] that has been overlooked in the recent literature on quantum nanophotonics. After presenting our design method, we assess the concurrence attained in the cloaks, revealing remarkable enhancements with respect to free space. Next, we analyze the dielectric spatial distribution within the devices, and offer insights into their performance by investigating the character and strength of the QE interactions as a function of the input parameters. Finally, we benchmark the degree of entanglement in our ID structures against those obtained using the negativity [42, 43] as the optimization function, and show that our designs yield steady-state concurrence values approaching the limit of maximally entangled-mixed-states [18, 19].

## 2 Physical system and design methodology

The system under study consists of a pair of distant QEs, modelled as identical two-level systems with perfect quantum yield, under incoherent pumping. Assuming that they are only weakly coupled to their dielectric environment, and after tracing out the EM degrees of freedom, the master equation describing the photon-assisted interactions between them (see Refs. [13, 44] for a complete derivation) has the form

(1) $$ı\left[\rho ,H\right]+\underset{i,j}{\sum }\frac{\gamma_{ij}}{2}L_{ij}\left(\rho \right)+\underset{i}{\sum }\frac{P_{i}}{2}L_{ii}^{\prime }\left(\rho \right)=0,$$

with $H=\sum_{i}\omega \sigma_{i}^{†}\sigma_{i}+\sum_{i\neq j}g_{ij}\sigma_{i}^{†}\sigma_{j}$ , and where the indices *i* = 1, 2 and *j* = 1, 2 label the two emitters. The first term in Eq. (1) accounts for the QE–QE coherent coupling, with *σ*_*i*_ $\left(\sigma_{i}^{†}\right)$ being the annihilation (creation) operator for the emitter *i*. The second one includes Lindblad superoperators of the form $L_{ij}\left(\rho \right)=2\sigma_{j}\rho \sigma_{i}^{†}-\sigma_{i}^{†}\sigma_{j}\rho -\rho \sigma_{i}^{†}\sigma_{j}$ and reflects the dissipative interaction between the QEs (*i* ≠ *j*), as well as their radiative decay (*i* = *j*). Finally, the incoherent pumping of both QEs is expressed in terms of Lindblad superoperators $L_{ii}^{\prime }\left(\rho \right)=2\sigma_{i}^{†}\rho \sigma_{i}-\sigma_{i}\sigma_{i}^{†}\rho -\rho \sigma_{i}\sigma_{i}^{†}$ . The analytical expression for the steady-state density matrix, *ρ*, solution of Eq. (1) can be found in the Supplementary Material [45].

There are four different sets of parameters in Eq. (1). First, the QEs natural frequency, *ω*, which we set to 3.1 eV (*λ* = 400 nm). Note that in this frequency range, metals sustain highly confined surface plasmon modes, which have been exploited recently in other nanophotonic proposals for entanglement generation [11], [12], [13, 46, 47]. Second, the incoherent pumping rate on each QE, which is assumed to be symmetric, *P*_*i*_ = *P*_*j*_ = *P*, and can be externally controlled by, for instance, optical or electrical means [40, 41]. Last, the coherent and dissipative coupling strengths, that can be expressed as a function of the Dyadic Green’s function **G**(**r**, **r′**, *ω*) [48] for the dielectric environment, evaluated at the QEs natural frequency. They read $g_{ij}=\omega^{2}Re\left\{p^{*}G\left(r_{i},r_{j},\omega \right)p\right\}/\hbar \varepsilon_{0}c^{2}$ and $\gamma_{ij}=2\omega^{2}Im\left\{p^{*}G\left(r_{i},r_{j},\omega \right)p\right\}/\hbar \varepsilon_{0}c^{2}$ , respectively, where **p** is the transition dipole moment of the QEs and **r**_*i*,*j*_, their position. In open non-chiral EM systems [49], the coupling constants (*i* ≠ *j*) fulfil *γ*_*ij*_ = *γ*_*ji*_ and *g*_*ij*_ = *g*_*ji*_. For *i* = *j*, *γ*_*ii*_ = *F*(*ω*, **r**_*i*_)*γ*_0_ gives the QE decay rate, where *F*(*ω*, **r**_*i*_) is the Purcell factor it experiences, and *γ*_0_ = *ω* ^3^| ***p*** |^2^/3*πℏɛ*_0_ *c* ^3^ its decay rate in free space.

With the density matrix, *ρ*, expressions for the expectation values of any physical observable for the system (or in our case, of an entanglement witness) can be constructed, which present an explicit dependence on the master equation parameters and, therefore, on the Dyadic Green’s function. Taking a given physical quantity as the target function, our ID approach seeks for the QEs dielectric environment (the spatial distribution of the permittivity around them) that optimizes (generally maximizes or minimizes) it. We follow a topology-optimization-inspired algorithm whose starting point is free space, i.e., *ϵ*_1_(**r**) = 1 in the whole domain of interest. The iterative procedure can be briefly described as follows: Each iteration step, labeled as *n*, consists in a spatial sweep around the QEs. At each position, **r**_*k*_ (of volume *δV*_*k*_), an small increment is introduced in the dielectric constant, $\epsilon_{n+1}^{\prime }\left(r_{k}\right)=\epsilon_{n}\left(r_{k}\right)+\delta \epsilon$ (note that, for clarity, we have introduced index *k* to reflect the spatial discretization of the permittivity map). This modifies the target function through the Dyadic Green’s function. If this local dielectric alteration contributes towards the optimization, then $\epsilon_{n+1}\left(r_{k}\right)=\epsilon_{n+1}^{\prime }\left(r_{k}\right)$ . Otherwise, the increment is discarded and *ϵ*_*n*+1_(**r**_*k*_) = *ϵ*_*n*_(**r**_*k*_).

In principle, the approach introduced above requires computing **G**(**r**_*i*_, **r**_*j*_, *ω*) for each local dielectric increment *k* and each iteration step *n*. This is, in general, largely computationally demanding. However, for small enough *δϵ*, the modification in the Dyadic Green’s function induced by the permittivity change at **r**_*k*_ can be described perturbatively. Thus, keeping only the first term in the born scattering series [34, 48], we have (see the Supplementary Material)

(2) $$\delta_{k}^{\prime }G_{n+1}\left(r_{i},r_{j},\omega \right)=\frac{\omega^{2}}{c^{2}}G_{n}\left(r_{i},r_{k},\omega \right)\delta \epsilon G_{n}\left(r_{k},r_{j},\omega \right)\delta V_{k},$$

whose effect in the target function still needs to be evaluated. If this local variation of the permittivity contributes to its optimization, *δϵ* is kept and $\delta_{k}G_{n+1}\left(r_{i},r_{j},\omega \right)=\delta_{k}^{\prime }G_{n+1}\left(r_{i},r_{j},\omega \right)$ , while *δϵ* is discarded and *δ*_*k*_ **G**_*n*+1_(**r**_*i*_, **r**_*j*_, *ω*) = 0 otherwise. As a result of the sweep in *k* a new, complete, permittivity map, *ϵ*_*n*+1_(**r**), is obtained, for which the Dyadic Green’s function **G**_*n*+1_(**r**_*i*_, **r**_*j*_, *ω*) can be calculated through EM simulations. Moreover, the convergence of the algorithm can be easily tested after each iteration step by computing

(3) $$G_{n+1}^{\prime }\left(r_{i},r_{j},\omega \right)=G_{n}\left(r_{i},r_{j},\omega \right)+\underset{k}{\sum }\delta_{k}G_{n+1}\left(r_{i},r_{j},\omega \right),$$

and verifying that $G_{n+1}^{\prime }\left(r_{i},r_{j},\omega \right)=G_{n+1}\left(r_{i},r_{j},\omega \right)$ within the accuracy preset for the algorithm. Importantly, using that $G_{n}\left(r_{k},r_{j},\omega \right)=G_{n}^{\text{T}}\left(r_{j},r_{k},\omega \right)$ , the evaluation of Eq. (2) in all space only requires two EM simulations. For the iteration *n* + 1, these correspond to the spatial profile of the electric fields radiated by both QEs, independently, within the permittivity map *ϵ*_*n*_(**r**).

**Figure 1:**
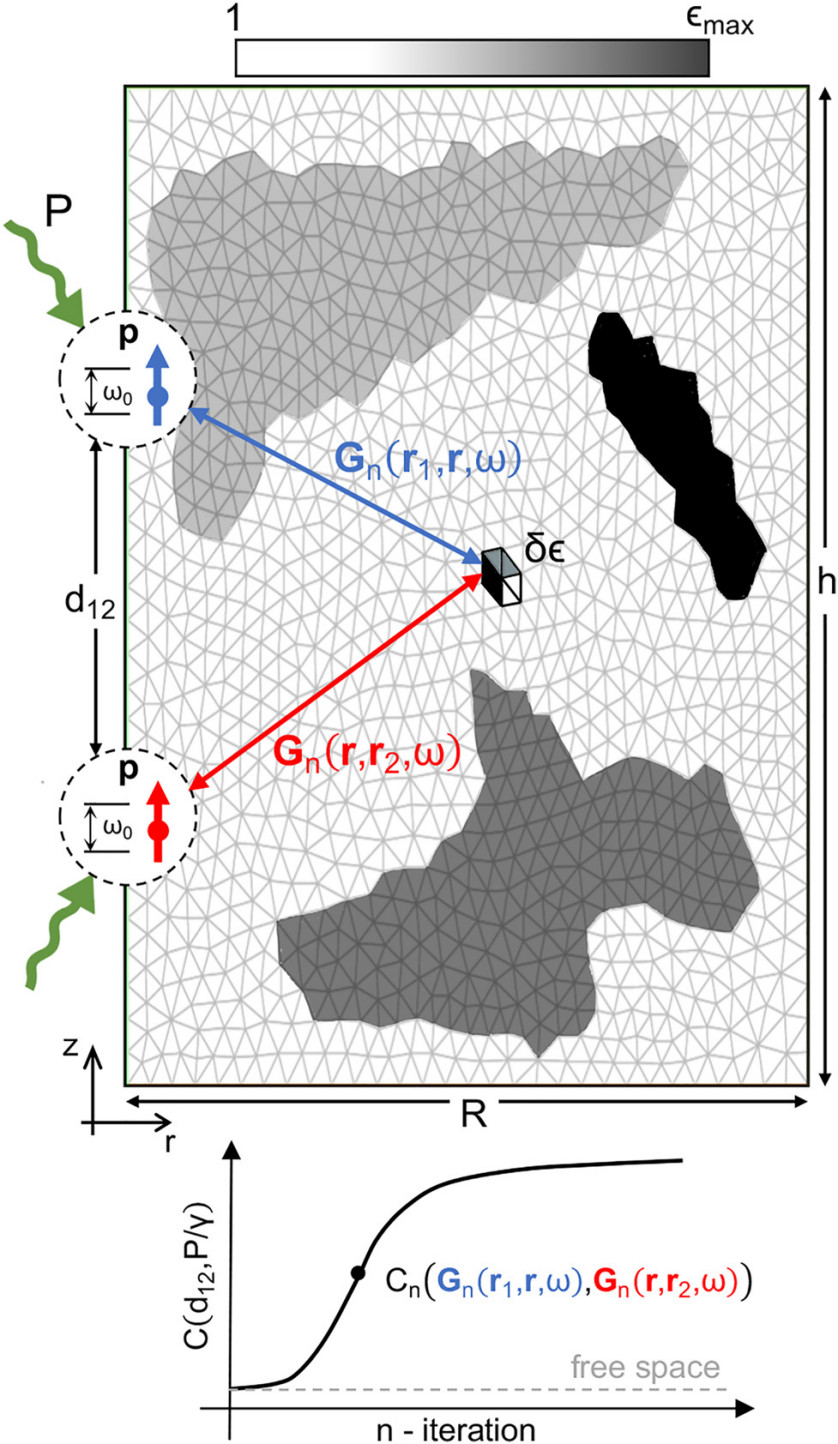
Description of the design algorithm performance. (a) Sketch of the topology-optimization design of a cylindrical cloak, of radius *R* and height *h*, that maximizes the Wootters concurrence between two QEs aligned along *z*-direction and separated by a distance *d*_12_. The light grey mesh renders the spatial discretization, while the device permittivity at iteration *n* is coded from white (*ϵ*_*n*_ = 1) to black (*ϵ*_*n*_ = *ϵ*_max_). The bottom panel illustrates the concurrence, *C*_*n*_ for the QE pair, as a function of the iteration step, *n*.

Figure 1 illustrates the ID approach described above. We employ the finite-element solver of Maxwell’s Equations implemented in Comsol Multiphysics™, whose spatial discretization is represented by the light gray thin mesh. Note that we employ this grid for the permittivity spatial distribution as well. In our designs, both QEs are aligned, with their dipole moments parallel to the axis that connects them (*z*-direction). This way, we can exploit the azimuthal symmetry of the system to solve the 3D EM problem within the *rz*-plane only. The size of the cylindrical cloaks is given by the parameters *R* and *h*, while the distance between the QEs is *d*_12_ (taken as an input parameter). *ϵ*_max_ is the maximum dielectric constant in the device, which varies from one design to another. In our calculations, we have set a threshold, *ϵ*_max_ ≤ 9, which corresponds to semiconductor materials such as GaP [50]. As anticipated, we take the Wootters concurrence as a measure of entanglement and therefore, as the optimization (in this case, maximization) function. This is defined in terms of the eigenvalues of the matrix ρTρ*T, where *T* is the anti-diagonal matrix with elements {−1, 1, 1, −1}. For our system, we have

(4) $$C=C\left(\rho \right)=2\;\max\left\{0,|\rho_{12}|-\sqrt{\rho_{00}\rho_{33}}\right\},$$

where *ρ*_00_ = ⟨*g*_1_ *g*_2_|*ρ*|*g*_1_ *g*_2_⟩ and *ρ*_33_ = ⟨*e*_1_ *e*_2_|*ρ*|*e*_1_ *e*_2_⟩ are the population of the ground and biexciton states and *ρ*_12_ = ⟨*e*_1_ *g*_2_|*ρ*|*g*_1_ *e*_2_⟩ is the coherence between single excitation states. A maximally entangled (completely untangled) state is characterized by *C* = 1 (*C* = 0). The lower panel of Figure 1 sketches the concurrence maximization, where *C*_*n*_ = *C*_*n*_(**G**_n_ (**r**_1_, ***r***, *ω*), **G**_n_(**r**, **r**_2_, *ω*)) corresponds to its value at iteration *n* (note that we have made explicit its dependence on the Dyadic Green’s function connecting the QE positions and the whole volume of the dielectric cloak). In the Supplementary Material, more details on different aspects of the topology-optimization method are provided, such as the convergence procedure and an assessment of the impact that the permittivity binarization has on the cloak performance. It also presents the expressions for the different parameters in Eq. (1) evaluated in free space. As part of the Supplemental Material, the codes and templates implementing our topology optimization approach are also provided.

## 3 Results

**Figure 2:**
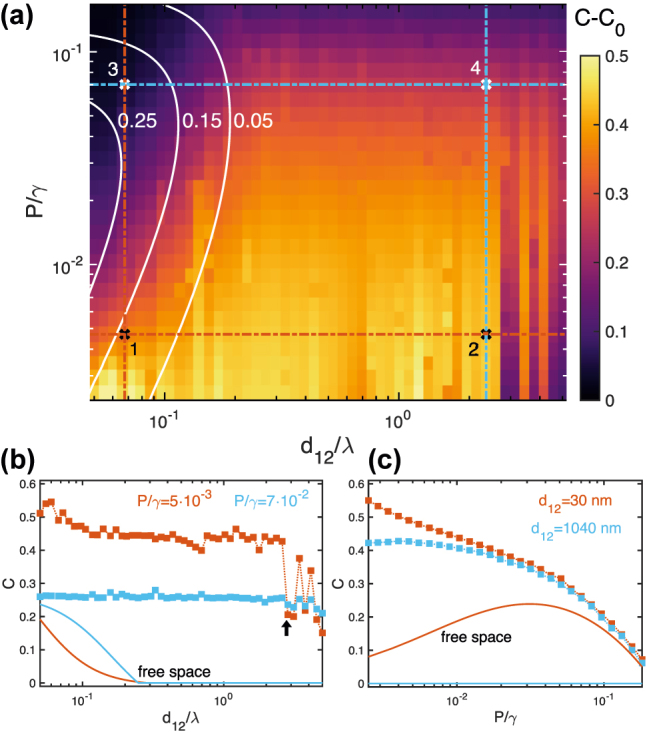
Concurrence as a function of distance and pumping strength. (a) Entanglement generation efficiency, *C* − *C*_0_, versus inter-emitter distance and pumping rate for 50 × 27 = 1350 ID cloaks. White solid lines correspond to QE–QE systems yielding three different values of the free-space concurrence, *C*_0_. Vertical and horizontal lines indicate the configurations considered in the panels below. (b) Cloak-induced (square dots) and free-space (solid lines) concurrences versus inter-emitter distance for low (orange) and high (blue) pumping. (c) Same as (b) but versus pumping strength and for short (orange) and long (blue) inter-emitter distance.

Figure 2 investigates the performance of the dielectric cloaks (with dimensions *R* = 3.75*λ* and *h* = 10*λ*) obtained through the topology-optimization procedure described above. The color map in Figure 2(a) displays *C* − *C*_0_, the difference between the QE–QE concurrence, *C*, for 1350 ID structures and their free-standing counterpart, *C*_0_ (obtained from the evaluation of Eq. (4) for free-space master equation parameters). This quantity, which we take as a measure of entanglement generation efficiency, is rendered against the inter-emitter distance, *d*_12_ (normalized to the QE wavelength, *λ* = 400 nm), and pumping strength, *P* (normalized to *γ* = *γ*_11_ = *γ*_22_, the emitter decay rate). In free space, *γ* = *γ*_0_, while *F*(*ω*, **r**_1,2_) ≠ 1 within the ID devices. Note that, although this is not a constraint imposed in our design strategy, both QEs experience the same Purcell factor, *F*(*ω*, **r**_1_) = *F*(*ω*, **r**_2_), in all the structures generated. Thus, the horizontal axis in Figure 2(a) sets the minimum optical path between the QEs, while the vertical one serves as a measure of their steady-state population ( $\rho_{11}^{\text{iso}}=P/\left(\gamma +P\right)$ for the QEs in isolation [51]). Both are in log scale, with a logarithmic density of system configurations as well. The white solid lines correspond to the pumping and distance conditions yielding three different values of *C*_0_: 0.25, 0.15 and 0.05. The latter can be identified as the boundary beyond which the free-space concurrence vanishes, as |*ρ*_12_| < *ρ*_00_ *ρ*_33_ in Eq. (4). Remarkably, it is exactly in this region where the dielectric cloaks perform best, leading to *C* − *C*_0_ = *C* ≈ 0.5 for distances up to 2.85*λ* = 1140 nm and low pumping rate. We anticipate here that this concurrence enhancement approaches the limit of maximum-entangled-mixed-states [18]. At smaller *d*_12_ and larger *P*, where *C*_0_ is not negligible, their efficiency worsens. This shows that, rather than enhancing *C*, our ID devices are able to generate entanglement in QE–QE configurations where the free-space concurrence vanishes. In the Supplementary Material, we present a map of the steady-state second-order cross-correlation function equivalent to Figure 2(a), which shows that, as expected [51], the entanglement enhancement induced by the dielectric structures also translates into an stronger antibunched light emission by the QEs.

Figure 2(b) analyzes the dependence of the entanglement generation efficiency on the inter-emitter distance for two different pumping rates. The square dots plot *C* as a function of *d*_12_/*λ* along the two horizontal lines indicated in panel (a). For comparison, *C*_0_ in the same pumping conditions are plotted in solid lines. We can observe that the cloak-induced concurrence presents little sensitivity to the QE–QE distance at high pumping (blue), and it decays slowly with distance at low *P* (orange). Both set of data present an abrupt reduction in *C* at *d*_12_ = 1140 nm (2.85*λ*, marked by a black vertical arrow) followed by oscillations, more apparent at low pumping. As shown below, these features originate from finite size effects, which become stronger as the inter-emitter distance approaches the cloak dimensions. In Figure 2(c), the effect of the pumping strength is explored. It plots *C* and *C*_0_ along the vertical lines in panel (a). The former decays monotonically with *P*/*λ* in a very similar way for the two distances considered. There exist differences at very small *P*, where the cloaks for short inter-emitter distance (orange) yield larger *C* than the ones for long distance (blue).

**Figure 3:**
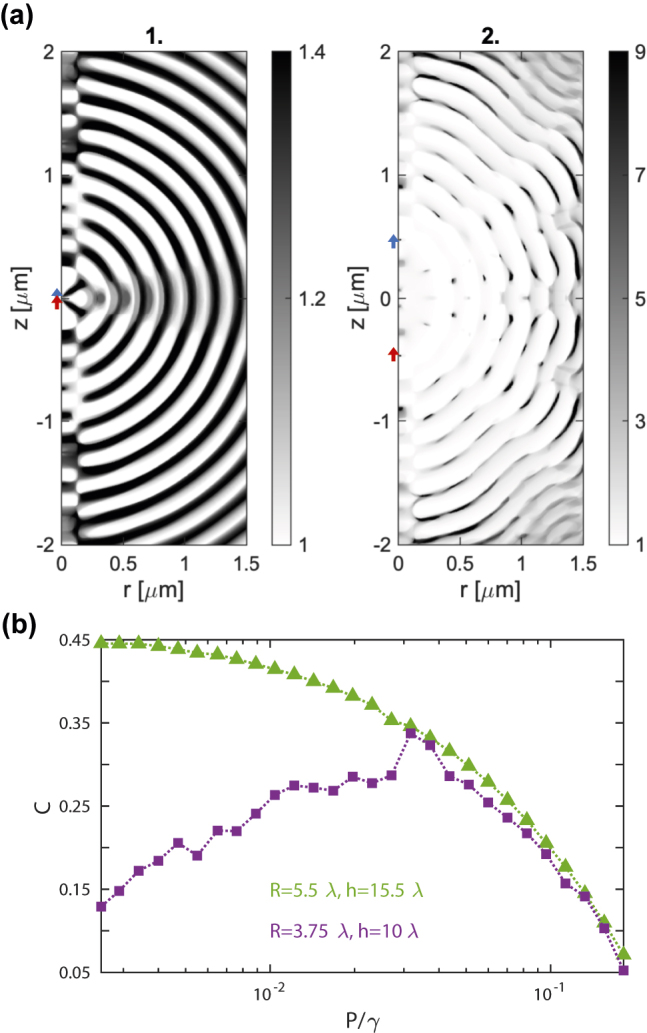
Dielectric cloaks and finite size effects. (a) Permittivity maps for the ID cloaks labeled as 1 (left) and 2 (right) in Figure 2(a). The dielectric constant is represented by white-to-black linear scales with different *ϵ*_max_. (b) Concurrence versus pumping rate for devices of two different sizes: *R* = 3.75*λ* and *h* = 10*λ* (purple) and *R* = 5.5*λ* and *h* = 15.5*λ* (green). Both set of cloaks operate at *d*_12_ = 2.85*λ*, QE–QE distance indicated by the vertical black arrow in Figure 2(b).

The dielectric distribution, *ϵ*(**r**), for the cloaks labeled as 1 and 2 in Figure 2(a) is shown in Figure 3(a). These are chosen in the low pumping regime (*P*/*γ* = 5 ⋅ 10^−3^), where *C* − *C*_0_ is largest. Note that exploiting the cylindrical symmetry of the designs, the permittivity maps are fully characterized within the *rz*-plane, with the QE positions indicated by red and blue arrows along the *z*-axis. The grey scale codes the dielectric constant linearly from 1 (white) to *ϵ*_max_ (black). In the left panel (1), the QEs are only a few nanometers apart (*d*_12_ = 30 nm), and the permittivity contrast is small, *ϵ*_max_ = 1.4. We can identify two different structures in the cloak. First, a narrow waveguide along *z*-direction, with radius *λ*/2 = 200 nm, approximately, that mediates the QE–QE interactions. Around it, a periodic and concentric pattern is apparent, with elements that act as reflectors that reduce radiation leakage. The dielectric distribution in this region is mainly binary, *ϵ*(**r**) = 1 or *ϵ*_max_, except around the *z* = 0 plane, along which dipole radiation power is maximum and the permittivity acquires intermediate values. The right panel of Figure 3(a) corresponds to device 2, the QEs are farther apart (*d*_12_ = 950 nm) and the maximum permittivity is much larger (*ϵ*_max_ = 9). This is the threshold value set for the topology-optimization algorithm, whose convergence required significantly more iterations than in the left panel. The resulting *ϵ*(**r**) still resembles device 1. The dielectric contrast along *z*-axis is now much smaller than around it. This is specially evident between the QEs. The geometry of the reflecting elements is more complex, with much sharper and isolated high-permittivity scatterers that overlap with multiple periodic-like patterns of moderate dielectric constant. In contrast to the left panel, the cloak is far from binary, with *ϵ*(**r**) varying smoothly in some spatial regions and much more abruptly in others. The underlying similarities between devices 1 and 2 in Figure 3(a) indicates that both ID cloaks generate entanglement by simultaneously engineering the mutual coupling between the QEs and minimizing their emission into free-space.

Figure 3(b) reveals the impact of the finite size of the ID cloaks in their performance. It plots the concurrence versus pumping strength for devices operating at *d*_12_ = 2.85*λ* = 1140 nm, the distance indicated by a black arrow in Figure 2(b). The purple dots correspond to the structures in that panel, with dimensions *R* = 3.75*λ* and *h* = 10*λ*. The green dots, to larger topology-optimized cloaks, with *R* = 5.5*λ* and *h* = 15.5*λ*. Both sets of data overlap at *P* > 3 ⋅ 10^−2^ *λ*. At lower pumping strengths, however, the concurrence decays significantly with decreasing *P* in the small devices, while it grows towards *C* ≈ 0.5 in the large ones. Importantly, the data for the latter resembles very much to those in Figure 2(c), which corresponded to smaller *d*_12_. The Supplementary Material displays the permittivity map for one of the large devices in Figure 3(b), and shows that it is not merely an extension of its smaller counterpart (it presents small differences in *ϵ*(**r**) in the vicinity of the QEs). Despite these near-field discrepancies, we can identify the reduction of entanglement in small cloaks with finite-size effects, as the number of reflecting elements in the cloaks is not enough to prevent the occurrence of significant radiation loss.

**Figure 4:**
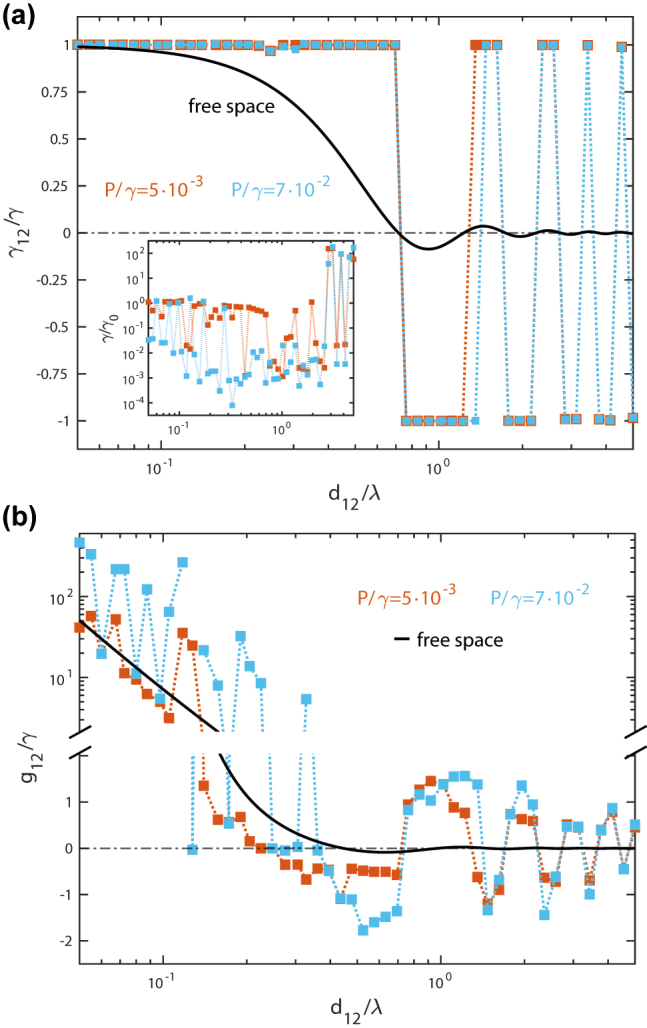
Optimized master equation parameters. (a) Dissipative coupling strength normalized to the QE decay rate as a function of the inter-emitter distance for the two pumping rates in Figure 2(b and c). The inset plots the purcell factor versus *d*_12_ for the cloaks in the main panel. (b) Normalized coherent coupling strength for the same ID structures. The black solid lines in both panels correspond to free-space magnitudes.

In order to shed light into the entanglement generation mechanism taking place in the ID cloaks, we examine the dissipative, *γ*_12_, and coherent, *g*_12_ coupling strengths that results from the topology-optimization design. Figure 4(a) plots the former, normalized to the QE decay rate, as a function of the inter-emitter distance and for the two pumping rates considered in Figure 2(b). For comparison, the same magnitude evaluated in free space is rendered in black solid line. We can observe that all the designs maximize the dissipative coupling, so that |*γ*_12_|/*γ* = 1, while its sign follows its free-standing counterpart. Note as well that the data for both *P*/*γ* overlap. These results demonstrate that the topology-optimized dielectric structures generate entanglement through the same dissipative-driven mechanism that occurs naturally in metal-based plasmonic nanostructures [11, 12, 46]. The inset of Figure 4(a) displays the Purcell factor, *F* = *γ*/*γ*_0_, experienced by both QEs for the designs in the main panel. It shows that the ID devices are capable of implementing the maximum dissipative coupling condition for extremely different QE decay rates. Remarkably, *γ*/*γ*_0_ ranges 6 orders of magnitude in the cloaks. On the one hand, *γ* is reduced up to a factor 10^−4^ for *d*_12_ < 3*λ*. On the other hand, it becomes 100-fold enhanced for larger inter-emitter distances, where the device efficiency diminishes due to finite size effects. Figure 4(b) displays the coherent coupling in the cloaks, revealing that they introduce only small deviations from free space. At small QE–QE distances, *g*_12_ ≫ *γ*, in the regime where the entanglement enhancement by the cloaks, *C* − *C*_0_, is moderate. On the contrary, |*g*_12_|/*γ* ≈ 1 at longer *d*_12_, where the coherent coupling vanish in free space.

Up to here, we have considered only the Wootters concurrence as a measure of entanglement. However, there exist multiple witnesses that have been proposed for bipartite systems [52]. Next, we take another, the (linear) negativity, *N* [42, 43], to assess the suitability of the Wootters concurrence, *C*, as the optimization function in our ID approach. The negativity is defined in terms of the negative eigenvalues of the partial transpose of the density matrix, *ρ*. For our system, it has a very simple form as well,

(5) $$\begin{array}{ll} N & =N\left(\rho \right)\\ & =\max\left\{0,\sqrt{{\left(\rho_{00}-\rho_{33}\right)}^{2}+4|\rho_{12}{|}^{2}}-\left(\rho_{00}+\rho_{33}\right)\right\}. \end{array}$$

**Figure 5:**
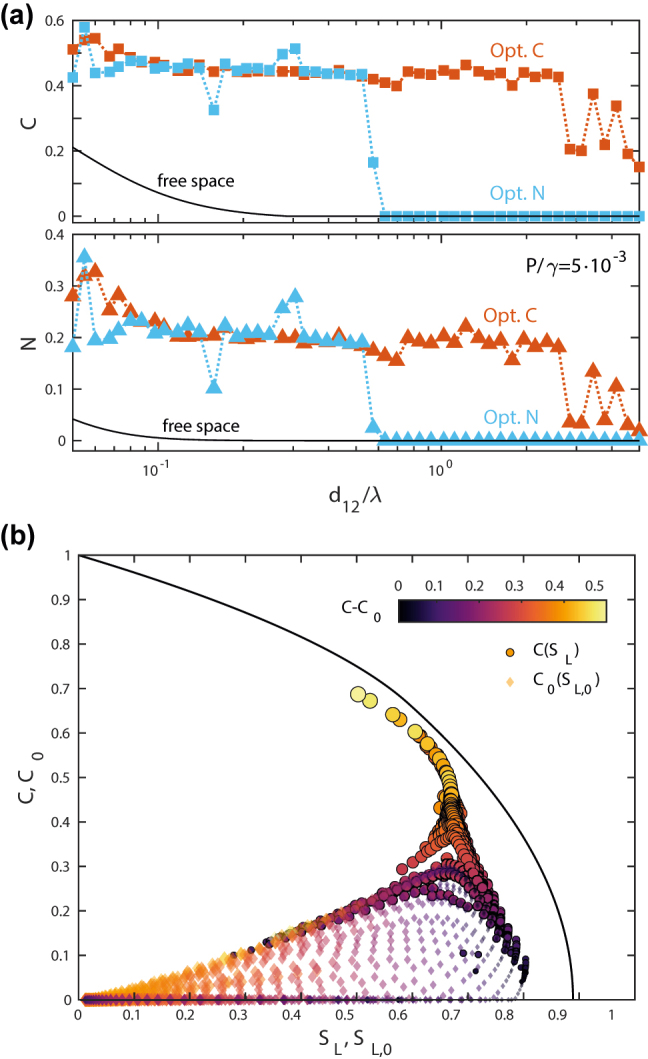
Concurrence, negativity and linear entropy. (a) Top: Concurrence versus inter-emitter distance for dielectric structures obtained by setting the concurrence (orange) and the negativity (blue) as the optimization function (*P*/*γ* = 5 ⋅ 10^−3^). Bottom: Negativity versus distance for the same designs as in the top panel. In both cases, black solid lines represent the results for free-standing QE pairs. (b) Concurrence versus linear entropy for the ID cloaks in Figure 2(a) (bright color circles), together with the corresponding values for the QE pairs in free space (faint color rhombuses). The colors code the concurrence enhancement, *C* − *C*_0_ for each system. The black solid line corresponds to the maximally entangled-mixed-states figure of merit.

By simple inspection, we can conclude that, similarly to Eq. (4), entanglement formation (*N* > 0) takes place under the condition |*ρ*_12_| > *ρ*_00_ *ρ*_33_ in Eq. (5). In Figure 5(a), we explore whether both equations also yield the same dielectric structures when employed as the maximization function in our topology-optimization algorithm. The top panel plots the concurrence versus inter-emitter distance for cloaks operating at *P*/*γ* = 5 ⋅ 10^−3^. Orange (blue) dots correspond to the designs obtained for concurrence (negativity) maximization, and the solid black lines plot *C*_0_. We can observe that for *d*_12_ ≲ *λ*/2 both sets of devices yield the same concurrence. The dielectric maps obtained from the maximization of both magnitudes are the same in this regime. On the contrary, at larger distances, the negativity-based algorithm does not find the optimization path in the concurrence-based procedure. This way, the outcome of the former is simply free space. To shed light into this finding, the lower panel of Figure 5(a) plots the negativity for the same structures, together with its free-space value, reproducing the same behavior. Note that *N* ≫ *N*_0_ at large distances only in the designs obtained by setting *C* as the optimization function. These results manifest that, as expected, our gradient-based topology-optimization approach is very sensitive not only to the target function, but also to the initial conditions (always set to free space in our study).

Finally, and once we have shown the dependence of the cloak designs on the optimization function, we proceed to benchmark their performance against maximally-entangled-mixed-states [18]. To do so, we calculate first the linear entropy for all the QE–QE states realized by the devices in Figure 2(a). This way, we establish their mixed/pure character. This quantity is defined in terms of the trace of the density matrix squared [42]. In our case, it reads $S_{L}=S_{L}\left(\rho \right)=\frac{4}{3}\left[1-\rho_{00}^{2}+\rho_{11}^{2}+\rho_{22}^{2}+\rho_{33}^{2}-2|\rho_{12}{|}^{2}\right]$ , being 0 for pure states, and 1 for maximally mixed ones. Figure 5(a) plots *C* versus *S*_L_ for our designs in bright color circles. Faint colored rhombuses render *C*_0_ as a function of *S*_L0_ for the same distance and pumping configurations but in free space. In both sets of data, the colors code the concurrence enhancement, *C* − *C*_0_, for each value of *d*_12_ and *P*. This panel shows clearly that our ID structures are most efficient when acting on QE–QE states that present a high purity in free space (*S*_L,0_ ≲ 0.4), while their impact is lower in free-standing states that present a higher entropy. This demonstrates that the designs enhance and generate entanglement by increasing the mixed character of the emitter states. In the Supplementary Material, the linear entropy enhancement map for our devices is presented, showing that *S*_L_ − *S*_L,0_ ∼ 0.6 for the designs yielding the maximum concurrence enhancement. Importantly, the black solid line in Figure 5(b) presents the concurrence-entropy curve for maximally entangled-mixed-states [19, 45]. It reveals clearly that the optimum cloaks approach greatly this figure of merit, yielding the maximum entanglement attainable for the linear entropy of the QE–QE state induced by the dielectric structure.

## 4 Conclusions

To conclude, we have applied inverse design ideas to the problem of bipartite entanglement generation under incoherent pumping conditions. Through a topology-optimization algorithm that, acting at the level of the electromagnetic Dyadic Green’s function, maximizes the Wootters concurrence, we have generated dielectric cloaks hosting quantum emitter pairs for different distance and pumping configurations. First, the entanglement enhancement provided by these devices has been assessed, showing that they are most efficient when operating on emitters that are completely untangled in free space. Next, we have analyzed the permittivity maps for these devices and explored the impact of finite-size effects in their performance. We have also shown that they operate by maximizing the dissipative coupling strength between the emitters, even under extremely different Purcell enhancement conditions. Finally, we have studied the dependence of the design outcome on the entanglement witness used as the optimization function, and have benchmarked our results against maximally-entangled-mixed-states. We believe that our work illustrates the power of inverse design as a tool to improve quantum information resources based on nanophotonic platforms, and opens the way towards the exploitation of similar approaches in larger, more complex, quantum emitter networks.

## Supplementary Material

Supplementary Material Details

Supplementary Material Details

Supplementary Material Details
