# Supplementary material for: Inverse-designed dielectric cloaks for entanglement generation
Source: Nanophotonics. 2022 Aug 22;11(19):4387–95. doi: 10.1515/nanoph-2022-0231 (PMC11501985; doi:10.1515/nanoph-2022-0231)
Supplement: Supplementary file 1 — Supplementary Material Details [file j_nanoph-2022-0231_suppl_001.pdf]

A. Miguel-Torcal, J. Abad-Arredondo, F. J. García-Vidal, and A. I. Fernández-Domínguez*

Supplementary Material: Inverse-designed dielectric cloaks for entanglement generation

Abstract: This Supplementary Material provides details on different aspects of the topology-optimization algorithm and the inverse-design (ID) cloaks presented in the main text. In Section 1, we introduce the solution for the steady-state density matrix for a pair of quantum emitters (QEs) under incoherent pumping, as well as analytical expressions for the concurrence and negativity of the system in terms of the parameters of the master equation. Section 2 describes the topology-optimization approach, with special emphasis on the Born series truncation employed to reduce its computational demands. In Section 3, the impact of finite-size effects in the ID structures presented in the main text is analyzed in detail. Next, Section 4 presents the analytical calculation of the linear entropy and the normalized second-order correlation function for cloaked and free-standing QE pairs. Finally, in Section 5 we introduce a binarization in the permittivity of our designs oriented to a possible experimental realization. We assess its impact on the performance of the cloaks.

1 Density matrix, concurrence and negativity

In order to obtain the density matrix in the steady state, solution to Equation (1) in the main text, we first project it into a basis for the vector space corresponding to the two identical two-level systems. The chosen basis is formed by the following states: $|0\rangle = |g_1 g_2\rangle$, $|1\rangle = |e_1 g_2\rangle$, $|2\rangle = |g_1 e_2\rangle$ and $|3\rangle = |e_1 e_2\rangle$, where $|g_i\rangle$ ($|e_i\rangle$) labels the ground (excited) state of qubit i. Then, we solve the set of linear equations and get a block diagonal density matrix [1] of the form,

$$\rho = \begin{pmatrix} \rho_{00} & 0 & 0 & 0 \\ 0 & \rho_{11} & \rho_{12} & 0 \\ 0 & \rho_{12}^* & \rho_{22} & 0 \\ 0 & 0 & 0 & \rho_{33} \end{pmatrix}, \tag{S1}$$

A. Miguel-Torcal, J. Abad-Arredondo, Departamento de Física Teórica de la Materia Condensada and Condensed Matter Physics Center (IFIMAC), Universidad Autónoma de Madrid, E- 28049 Madrid, Spain, alberto.miguel@uam.es https://orcid.org/0000-0003-4785-6079 (AMT), jaime.abad@uam.es https://orcid.org/0000-0003-3980-966X (JAA)
F. J. García-Vidal, Departamento de Física Teórica de la Materia Condensada and Condensed Matter Physics Center (IFIMAC), Universidad Autónoma de Madrid, E- 28049 Madrid, Spain, and Institute of High Performance Computing, Agency for Science, Technology, and Research (A*STAR), Connexis, 138632 Singapore, fj.garcia@uam.es https://orcid.org/0000-0003-4354-0982
***Corresponding author: A. I. Fernández-Domínguez,** Departamento de Física Teórica de la Materia Condensada and Condensed Matter Physics Center (IFIMAC), Universidad Autónoma de Madrid, E- 28049 Madrid, Spain, a.fernandez-dominguez@uam.es https://orcid.org/0000-0002-8082-395X

with

$$\rho_{11} = \frac{2r(1+r)\left(8\tilde{g}_{12}^2 + \tilde{\gamma}_2(1+r)^2\right)}{4(r-1)\tilde{\gamma}_{12}^2\left[16\tilde{g}_{12}^2 + (1+r)^2\right] + (1+r)^3\left[16\tilde{g}_{12}^2 + (4\tilde{\gamma}_1\tilde{\gamma}_2 + r(2+r))\right]}, \tag{S2a}$$

$$\rho_{22} = \frac{2r(1+r)\left(8\tilde{g}_{12}^2 + \tilde{\gamma}_1(1+r)^2\right)}{4(r-1)\tilde{\gamma}_{12}^2\left[16\tilde{g}_{12}^2 + (1+r)^2\right] + (1+r)^3\left[16\tilde{g}_{12}^2 + (4\tilde{\gamma}_1\tilde{\gamma}_2 + r(2+r))\right]}, \tag{S2b}$$

$$\rho_{33} = \frac{r}{2}(\rho_{11} + \rho_{22}), \tag{S2c}$$

$$\rho_{00} = 1 - \rho_{11} - \rho_{22} - \rho_{33}, \tag{S2d}$$

$$\rho_{12} = \frac{2i\tilde{g}_{12}(\rho_{11} - \rho_{22}) - \tilde{\gamma}_{12}(\rho_{11} + \rho_{22} - 2\rho_{33})}{1+r}. \tag{S2e}$$

The matrix entries above are expressed in terms of the normalized coupling strengths, $\tilde{g}_{12} = g_{12}/\Gamma$ and $\tilde{\gamma}_{12} = \gamma_{12}/\Gamma$, the normalized self-damping decay rates $\tilde{\gamma}_i = \gamma_i/\Gamma$ $(i = \{1,2\})$ and the normalized pumping $r = 2P/\Gamma$, where $\Gamma = \gamma_1 + \gamma_2$.

The analytical solution for the density matrix allows us to write the quantities chosen to quantify entanglement in the QE pair system in terms of the master equation parameters, the concurrence,

$$C = 2\ \max\left\{0, \sqrt{4\tilde{g}_{12}^2\left[r(1+r)^2\chi\right]^2 + \tilde{\gamma}_{12}^2\left[r(1-r)\xi\right]^2} - \sqrt{\frac{r^2(1+r)\xi}{2}\left[1 - r(2+r)(1+r)\xi\right]}\right\}, \tag{S3}$$

and the negativity,

$$N = \max\left\{0, \sqrt{\left[1 - r(1+r)^2\xi\right]^2 + 16\tilde{g}_{12}^2\left[r(1+r)^2\chi\right]^2 + 4\tilde{\gamma}_{12}^2\left[r(1-r)\xi\right]^2} + \left[1 - r(1+r)\xi\right]\right\}, \tag{S4}$$

where

$$\chi = \frac{2(\tilde{\gamma}_2 - \tilde{\gamma}_1)}{4(r-1)\tilde{\gamma}_{12}^2\left[16\tilde{g}_{12}^2 + (1+r)^2\right] + (1+r)^3\left[16\tilde{g}_{12}^2 + (4\tilde{\gamma}_1\tilde{\gamma}_2 + r(2+r))\right]} \tag{S5}$$

$$\xi = \frac{2\left(16\tilde{g}_{12}^2 + (1+r)^2\right)}{4(r-1)\tilde{\gamma}_{12}^2\left[16\tilde{g}_{12}^2 + (1+r)^2\right] + (1+r)^3\left[16\tilde{g}_{12}^2 + (4\tilde{\gamma}_1\tilde{\gamma}_2 + r(2+r))\right]}. \tag{S6}$$

Note that the concurrence and negativity in free space, C_0 and N_0, respectively (which also correspond to the initial step $\epsilon_1(\mathbf{r}_k) = 1$ in the optimization procedure) do not depend on the normalized coherent coupling strength, $\tilde{g}_{12}$. The electromagnetic Dyadic Green's function in free space yield $\gamma_1 = \gamma_2 \equiv \gamma$, and thus, $\chi = 0$ and $\xi = 2/\left[4(r-1)\tilde{\gamma}_{12}^2 + (1+r)^3\right]$. The coupling strengths evaluated in free space have the following analytical expressions [2],

$$\frac{g_{12}}{\gamma} = \frac{3}{2(2\pi\frac{d_{12}}{\lambda})^2}\left[\frac{\cos\left(2\pi\frac{d_{12}}{\lambda}\right)}{(2\pi\frac{d_{12}}{\lambda})} + \sin\left(2\pi\frac{d_{12}}{\lambda}\right)\right], \tag{S7}$$

and

$$\frac{\gamma_{12}}{\gamma} = \frac{3}{(2\pi\frac{d_{12}}{\lambda})^2}\left[\frac{\sin\left(2\pi\frac{d_{12}}{\lambda}\right)}{(2\pi\frac{d_{12}}{\lambda})} - \cos\left(2\pi\frac{d_{12}}{\lambda}\right)\right]. \tag{S8}$$

Let us remind here that these expressions correspond to two QEs oriented parallel to the vector that connects their position in free space.

2 Topology-optimization algorithm and Born series truncation

This section describes in detail the topology-optimization method, with special emphasis in the Dyadic Green's function, and provides different aspects of the entanglement generation process [3]. The Dyadic

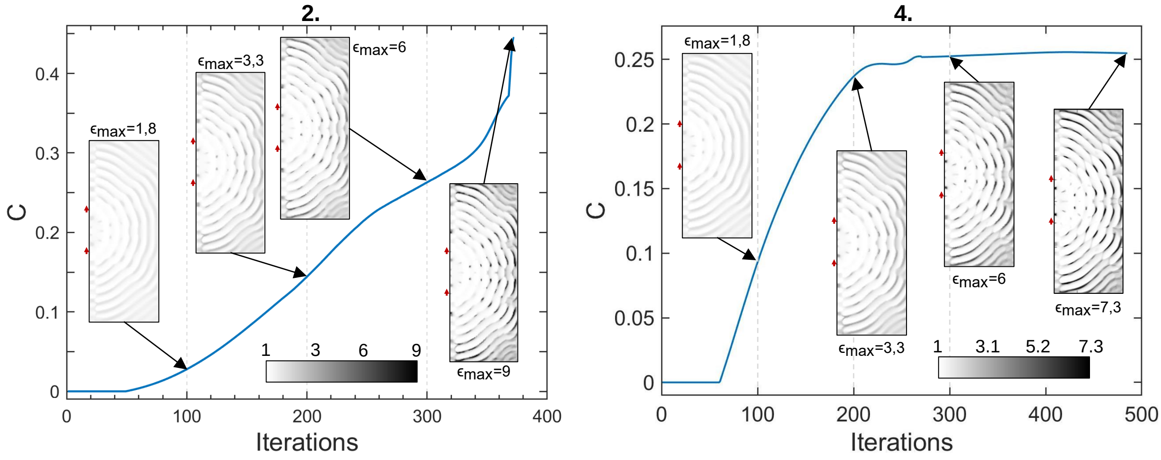

Fig. S1: Topology optimization procedures for the dielectric cloaks operating at $d_{12} = 950\,\text{nm}$ and $P = 5\cdot 10^{-3}\gamma$ ($P = 7\cdot 10^{-2}\gamma$), points 2 (left) and 4 (right) in Fig. 2(a) of the main text. The main panels plot concurrence versus iteration step. The insets display the permittivity map at four particular steps, $n = 100$, 200, 300 and 374 (485). The permittivity is rendered from white ($\epsilon = 1$) to black ($\epsilon = \epsilon_{\max}$) in linear scale. Note that the threshold $\epsilon_{\max} = 9$ is reached in the left panel at $n = 374$.

Green's function associated with the permittivity map $\epsilon'_{n+1}(\mathbf{r})$ in iteration step n is defined as the solution to the differential equation,

$$\nabla \times \nabla \times \mathbf{G}'_{n+1}(\mathbf{r}, \underline{\mathbf{r}}, \omega) - \frac{\omega^2}{c^2}\epsilon'_{n+1}(\mathbf{r})\mathbf{G}'_{n+1}(\mathbf{r}, \underline{\mathbf{r}}, \omega) = \mathbb{1}\,\delta(\mathbf{r} - \underline{\mathbf{r}})\,, \tag{S9}$$

where the spatial coordinates can be interpreted as follows: $\mathbf{r}$ indicates the point at which EM fields are evaluated while $\underline{\mathbf{r}}$ is the current source location. Note that the primes indicate that the Green's function and permittivity map are still unknown. To construct $\epsilon'_{n+1}(\mathbf{r})$, we decompose it into a 'background' part, given by the permittivity map at the previous iteration step and for which $\mathbf{G}_n(\mathbf{r}, \underline{\mathbf{r}}, \omega)$ is known, plus the small perturbation proportional to $\delta\epsilon$. Here, for clarity and in accordance with the main text, we employ a discrete description of this second term,

$$\epsilon'_{n+1}(\mathbf{r}) = \epsilon_n(\mathbf{r}) + \delta\epsilon \sum_k \delta(\mathbf{r} - \mathbf{r}_k)\delta V_k, \tag{S10}$$

where index k runs over all the discrete spatial positions, of volume δV_k, where the optimization algorithm introduces a permittivity alteration. Note that $\epsilon_n(\mathbf{r})$ is unprimed as it is the result of the previous optimization step.

Using the differential equation for the previous iteration (note the absence of primes as the Dyadic Green's function is known),

$$\nabla \times \nabla \times \mathbf{G}_n(\mathbf{r}, \underline{\mathbf{r}}, \omega) - \frac{\omega^2}{c^2}\epsilon_n(\mathbf{r})\mathbf{G}_n(\mathbf{r}, \underline{\mathbf{r}}, \omega) = \mathbb{1}\,\delta(\mathbf{r} - \underline{\mathbf{r}})\,, \tag{S11}$$

and Equation S10, we can rearrange Equation S9 as

$$\nabla \times \nabla \times \mathbf{G}'_{n+1}(\mathbf{r}, \underline{\mathbf{r}}, \omega) - \frac{\omega^2}{c^2}\epsilon_n(\mathbf{r})\mathbf{G}'_{n+1}(\mathbf{r}, \underline{\mathbf{r}}, \omega) = \mathbb{1}\,\delta(\mathbf{r} - \underline{\mathbf{r}}) + \sum_k \frac{\omega^2}{c^2}\,\delta\epsilon\,\mathbf{G}'_{n+1}(\mathbf{r}, \underline{\mathbf{r}}, \omega)\delta(\mathbf{r} - \mathbf{r}_k)\delta V_k. \tag{S12}$$

The formal solution to this equation reads [3]

$$\mathbf{G}'_{n+1}(\mathbf{r}, \underline{\mathbf{r}}, \omega) = \mathbf{G}_n(\mathbf{r}, \underline{\mathbf{r}}, \omega) + \frac{\omega^2}{c^2}\sum_k \delta\epsilon\,\mathbf{G}_n(\mathbf{r}, \mathbf{r}_k, \omega)\mathbf{G}'_{n+1}(\mathbf{r}_k, \underline{\mathbf{r}}, \omega)\delta V_k. \tag{S13}$$

Using that $\mathbf{G}_n(\mathbf{r}, \underline{\mathbf{r}}, \omega)$ satisfies Equation S11, this formal solution can be verified by direct substitution back into Equation S12. Equation S13 is exact, but infinitely recursive as $\mathbf{G}'_{n+1}(\mathbf{r}, \underline{\mathbf{r}}, \omega)$ appears on both

sides. Thus, by repeated use of Equation S13 one can write an iterative solution in powers of $\delta\epsilon$, known as Born series expansion

$$\mathbf{G}'_{n+1}(\mathbf{r},\underline{\mathbf{r}},\omega) = \mathbf{G}_n(\mathbf{r},\underline{\mathbf{r}},\omega) + \frac{\omega^2}{c^2}\sum_k \mathbf{G}_n(\mathbf{r},\mathbf{r}_k,\omega)\,\delta\epsilon\,\mathbf{G}_n(\mathbf{r}_k,\underline{\mathbf{r}},\omega)\delta V_k$$
$$+\left(\frac{\omega^2}{c^2}\right)^2 \sum_{k_1}\sum_{k_2} \mathbf{G}_n(\mathbf{r},\mathbf{r}_{k_1},\omega)\,\delta\epsilon_1\,\mathbf{G}_n(\mathbf{r}_{k_1},\mathbf{r}_{k_2},\omega)\,\delta\epsilon_2\,\mathbf{G}_n(\mathbf{r}_{k_2},\underline{\mathbf{r}},\omega)\delta V_{k_1}\delta V_{k_2} + \cdots . \tag{S14}$$

Keeping only the first term in the expansion and identifying $\delta'\mathbf{G}_{n+1} = \mathbf{G}'_{n+1} - \mathbf{G}_n$, one is left with

$$\delta'\mathbf{G}_{n+1}(\mathbf{r},\underline{\mathbf{r}},\omega) = \sum_k \delta'_k\mathbf{G}_{n+1}(\mathbf{r},\underline{\mathbf{r}},\omega) = \frac{\omega^2}{c^2}\sum_k \mathbf{G}_n(\mathbf{r},\mathbf{r}_k,\omega)\,\delta\epsilon\,\mathbf{G}_n(\mathbf{r}_k,\underline{\mathbf{r}},\omega)\delta V_k, \tag{S15}$$

and

$$\delta'_k\mathbf{G}_{n+1}(\mathbf{r},\underline{\mathbf{r}},\omega) = \frac{\omega^2}{c^2}\mathbf{G}_n(\mathbf{r},\mathbf{r}_k,\omega)\,\delta\epsilon\,\mathbf{G}_n(\mathbf{r}_k,\underline{\mathbf{r}},\omega)\delta V_k, \tag{S16}$$

which is identical to Equation (2) in the main text. Since the Born series in Equation S15 is truncated in the first term, it is clear that the perturbation in the permittivity must be small and localized enough to ensure the validity of the approximation ($\delta\epsilon\delta V_k \to 0$).

The first-order term in Equation S15, which requires only two EM simulations to be evaluated, allows us to calculate the permittivity-induced alteration of the QE-QE coherent and dissipative coupling strengths, as well as their radiative decay rates. Their changes due to $\delta\epsilon$ at position k and iteration n read

$$\delta'_{k,n}g_{ij} = \frac{\omega^4}{\hbar\epsilon_0 c^4}\,\mathrm{Re}\{\mathbf{p}^*\mathbf{G}_n(\mathbf{r}_i,\mathbf{r}_k,\omega)\,\delta\epsilon\,\delta V_k\mathbf{G}_n(\mathbf{r}_k,\mathbf{r}_j,\omega)\mathbf{p}\} \tag{S17}$$

and

$$\delta'_{k,n}\gamma_{ij} = \frac{2\omega^4}{\hbar\epsilon_0 c^4}\,\mathrm{Im}\{\mathbf{p}^*\mathbf{G}_n(\mathbf{r}_i,\mathbf{r}_k,\omega)\,\delta\epsilon\,\delta V_k\mathbf{G}_n(\mathbf{r}_k,\mathbf{r}_j,\omega)\mathbf{p}\}. \tag{S18}$$

In the equations above, we use the expressions for the parameters of the master equation in terms of the Dyadic Green's function presented in the main text. Taking into account that $\mathbf{G}_n(\mathbf{r}_i,\mathbf{r}_k,\omega) = \mathbf{G}_n^T(\mathbf{r}_k,\mathbf{r}_i,\omega)$, the dependence of Equation S17 and Equation S18 on the simulated electric fields obtained for both QEs radiating, independently, within the dielectric permittivity map can be written as

$$\delta'_{k,n}g_{ij} = \frac{\epsilon_0}{\hbar}\,\mathrm{Re}\big\{\delta\epsilon\,\delta V_k\mathbf{E}^T_{i,n}(\mathbf{r}_k)\mathbf{E}_{j,n}(\mathbf{r}_k)\big\}, \tag{S19}$$
$$\delta'_{k,n}\gamma_{ij} = \frac{2\epsilon_0}{\hbar}\,\mathrm{Im}\big\{\delta\epsilon\delta V_k\mathbf{E}^T_{i,n}(\mathbf{r}_k)\mathbf{E}_{j,n}(\mathbf{r}_k)\big\}, \tag{S20}$$

where $\mathbf{E}_{i,n}(\mathbf{r}_k) = \frac{\omega^2}{\epsilon_0 c^2}\mathbf{G}_n(\mathbf{r}_k,\mathbf{r}_i,\omega)\mathbf{p}_i$ are the radiated fields by QE i within the permittivity map $\epsilon_n(\mathbf{r})$.

Using Equation S19 and Equation S20, we can evaluated the effect of the localized permittivity alteration in the target function, which depends, in general, on the master equation parameters $f = f(\gamma_{ij}, g_{ij})$. In our designs, we aim to maximize the Wootters concurrence, $f \equiv C$, and therefore, we can write

$$\delta_{k,n}C = \left(\frac{\partial C}{\partial g_{12}}\right)\delta_{k,n}g_{12} + \left(\frac{\partial C}{\partial \gamma_{12}}\right)\delta_{k,n}\gamma_{12} + \left(\frac{\partial C}{\partial \gamma_1}\right)\delta_{k,n}\gamma_1 + \left(\frac{\partial C}{\partial \gamma_2}\right)\delta_{k,n}\gamma_2, \tag{S21}$$

which establishes the optimization criterion. During the iteration n, $\delta\epsilon$ is introduced at position $\mathbf{r}_k$ if $\delta_{k,n}C > 0$, while it is discarded otherwise. Thus, we have $\epsilon_{n+1}(\mathbf{r}_k) = \epsilon_n(\mathbf{r}_k) + \delta\epsilon$, while $\epsilon_{n+1}(\mathbf{r}_k) = \epsilon_n(\mathbf{r}_k)$ otherwise:

$$\epsilon_{n+1}(\mathbf{r}_k) = \begin{cases} \epsilon_n(\mathbf{r}_k) + \delta\epsilon & \text{if } \delta_{k,n}C > 0, \\ \epsilon_n(\mathbf{r}_k) & \text{otherwise.} \end{cases} \tag{S22}$$

This is the core of the topology-optimization algorithm, acting at the level of the electromagnetic Dyadic Green's function, employed for the design of the dielectric cloaks presented and investigated in the main text.

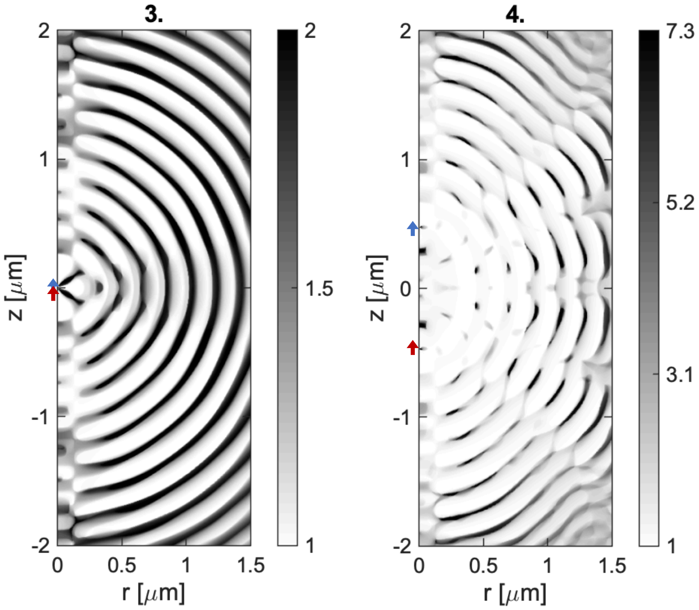

Fig. S2: Permittivity maps for the ID cloaks labelled as 3 (left) and 4 (right) in Figure 2(a). In the former, $d_{12} = 30\,\text{nm}$, in the latter, $d_{12} = 950\,\text{nm}$, while $P = 7 \cdot 10^{-2}\gamma$ in both panels. The dielectric constant is represented by white-to-black linear scales with different ϵ_{max}.

Figure S1 presents the topology-optimization process for points 2 and 4 in Figure 2(a) in the main text ($d_{12} = 950\,\text{nm}$, $P = 5 \cdot 10^{-3}\gamma$ and $P = 7 \cdot 10^{-2}\gamma$, respectively). It plots the concurrence versus iteration step showing the two possible ending mechanisms of the algorithm. It is set to end either if a plateau of concurrence values is found, as in the case on the right panel (last iteration at $n = 485$), or when the pre-set threshold for the dielectric constant, $\epsilon_{\text{max}} = 9$ (see main text), is reached (see left panel, with last iteration at $n = 374$). The permittivity maps at four specific steps, $n = 100$, 200, 300 and 374 (485) are also shown.

Figure S2 renders the permittivity maps for the ID cloaks at points 3 and 4 in Figure 2(a). These are taken in the high pumping regime ($P/\gamma = 7 \cdot 10^{-2}$), where the efficiency of entanglement generation worsens. The grey scale codes the dielectric constant linearly from 1 (white) to ϵ_{max} (black, different in each case). The map labelled as 3 (left) belongs to the region where both C_0 and $S_{L,0}$ are non-negligible. The algorithm does not find the optimization path and the generated structure does not provide a significant concurrence enhancement over free space. The dielectric map on the right resembles its low pumping counterpart, see map 2 in Figure 3(a). However, the design does not reach the threshold $\epsilon_{\text{max}} = 9$ and yields lower concurrence values. Note that, again, the initial state of the QEs in free space is partially mixed.

3 Finite-size effects

In the main text, we have shown that finite-size effects are relevant for ID cloaks operating at inter-emitter separations, d_{12}, comparable with the distance between the QE positions and the closest device edge (R and $\pm h/2$). This finding is illustrated in Figure 2, which corresponds $R = 3.75\lambda$ and $h = 10\lambda$. At $d_{12} \geq 3\lambda$, the concurrence enhancement significantly decreases and starts to oscillate. Figure 3(b) sheds insights into this result, and reveals a strong dependence on the pumping strength. Figure S3 completes this analysis by displaying concurrence versus pumping strength for devices operating at four different QE-QE distances at which the cloak performance drops. The purple dots correspond to structures with dimensions $R = 3.75\lambda$ and $h = 10\lambda$ while the green dots belong to larger topology-optimized cloaks, with $R = 5.5\lambda$ and $h = 15.5\lambda$ (the same sizes as those considered in Figure 3(b) of the main text). We can observe that the performance

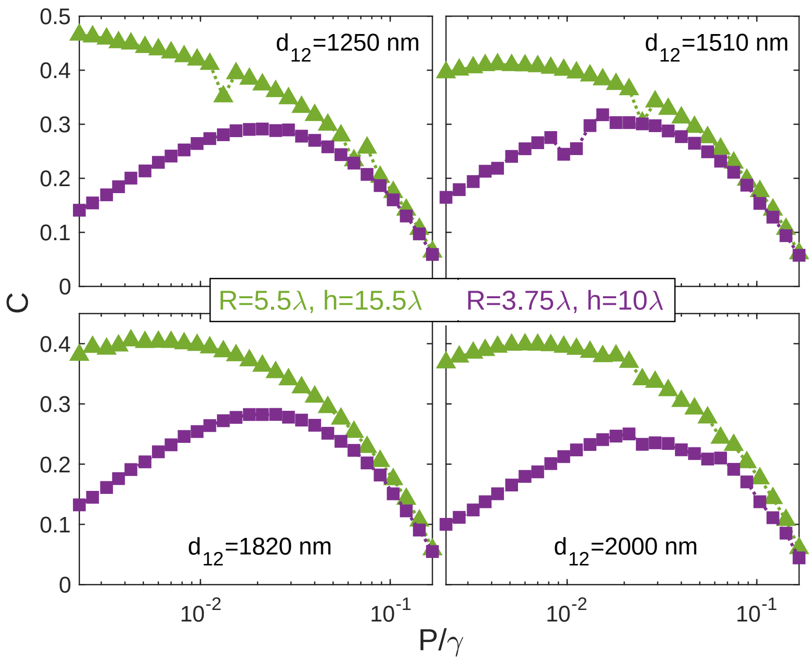

Fig. S3: Concurrence versus pumping rate for the two devices considered in Figure 3(b) of the main text and larger inter-emitter distances, d_{12}. The colors label the dimensions of the dielectric structure: $R = 5.5\lambda$, $h = 15.5\lambda$ (green) and $R = 3.75\lambda$, $h = 10\lambda$ (violet).

of the former barely depends on d_{12}, which indicates that, except for an slight reduction for all P, finite-size effects are similar for distances between 1250 and 2000 nm (3.1λ and 5λ, respectively). On the contrary, their impact in the larger devices increases for larger d_{12}. This is only apparent at very low pumping rates, where the devices perform best. The concurrence curve bends down in this region for $d_{12} > 1250$ nm.

The dielectric maps displayed in Figure S4 correspond to the devices operating at $d_{12} = 1250$ nm and $P/\gamma = 5 \cdot 10^{-3}$ in the top left panel of Figure S3. Apparently, the permittivity map on the left ($R = 5.5\lambda$, $h = 15.5\lambda$), is an extension of the right one ($R = 3.75\lambda$, $h = 10\lambda$). Indeed, both dielectric cloaks present a very similar structure in the region spanned by the smaller one. We can identify the waveguide elements along z-axis (see main text), as well as the lateral arrangements of sharp reflectors cancelling the emission into free space. As expected, in the large device, the topology-optimization procedure introduces more reflectors, which reduce radiation leakage more efficiently. However, a closer look into the dielectric distribution in the vicinity of the QEs reveals remarkable differences between the designs that are not so apparent initially. The zoom-in panels on the right hand side seem to indicate that the near-field maps promote radiation along the r-axis (z-axis) in the large (small) cloaks. Note the regions of large permittivity (in black) in each case. This indicates that the presence of extra reflectors in the larger design, and therefore the reduction of radiation loss, affects the permittivity in the vicinity of the QE. We can conclude that the topology-optimization procedure allows harnessing the radiation of the QEs along the radial direction for entanglement generation.

4 Linear entropy and second-order correlation function

As anticipated in the main text, the ID cloaks provide entanglement to the QE-QE states by increasing as well their mixed character, approaching the limit of maximum-entangled-mixed-states [1]. To show this more clearly, Figure S5 presents the linear entropy enhancement (defined as $S_L - S_{L,0}$, see main text) map for the devices in Figure 2. The resemblance with Figure 2(a) is remarkable. Both, the concurrence and entropy enhancements present the same structure, increasing for lower P and all inter-emitter distances

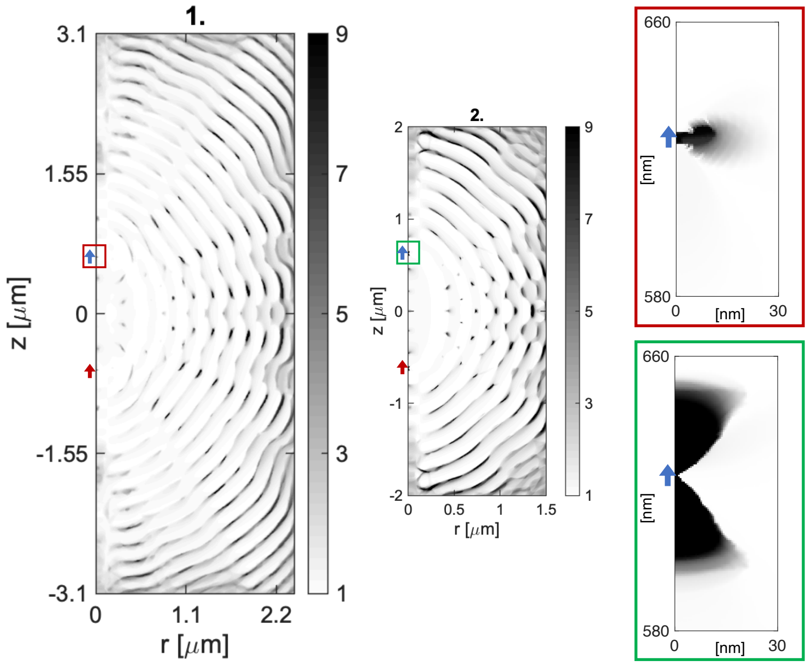

Fig. S4: Dielectric maps for ID cloaks of different dimensions: $R = 5.5\lambda$, $h = 15.5\lambda$ (left) and $R = 3.75\lambda$, $h = 10\lambda$ (right). The panels labelled as 1 and 2 correspond to devices operating at $d_{12} = 1250\,\text{nm}$ and $P = 5 \cdot 10^3\gamma$. The enlarged areas on the right panels zoom in the region close to the QEs, marked by red (1) and green (2) rectangles.

up to the region where finite-size effects come in. We can observe that the topology-optimized designs yield entropy enhancements as large as $S_L - S_{L,0} \approx 0.6$, the region where their performance approach the maximum-entangled-mixed-states curve in Figure 5(b).

Our work focuses on the entanglement generation in QE pairs by means of their dielectric environment. Thus, we have focused our attention in magnitudes such as the concurrence or negativity, which we employ as a measurement of the performance of the devices. Moreover, we have shed light into our findings by means of quantities such as the master equation parameters or the linear entropy. Here, we analyze briefly the properties of the light radiated by these systems, which can be used as an far-field witness of entanglement. In particular, we examine the zero-delay, normalized, second-order cross correlation function in the steady state. This gives us insight into the statistical properties of the photons emitted by the ID systems. In terms of the density matrix entries, it takes the form [4]

$$g_{12}^{(2)}(0) = \frac{\rho_{33}}{(\rho_{11} + \rho_{33})(\rho_{22} + \rho_{33})}. \tag{S23}$$

$g_{12}^{(2)}(0)$ is equal to one if the emitters act as uncorrelated, coherent, photon sources. On the contrary, it different from one if the emission from one QE is conditioned by the emission of the other QE. Indeed, the numerator above, ρ_{33}, is related to the probability of simultaneous photon emission (and detection), while the two factors in the denominator, $(\rho_{11} + \rho_{33})$ and $(\rho_{22} + \rho_{33})$, can be interpreted as the intensity of the light emitted from each QE. In terms of the normalized QE decay rates and coupling strengths, Equation S23 reads

$$g_{12}^{(2)}(0) = \left[\frac{r(1+r)(2+r)}{\mathcal{D}}(16\tilde{g}_{12}^2 + (1+r)^2) + \frac{4(1+r)(8\tilde{g}_{12}^2 + \tilde{\gamma}_1(1+r)^2)(8\tilde{g}_{12}^2 + \tilde{\gamma}_2(1+r)^2)}{\mathcal{D}(16\tilde{g}_{12}^2 + (1+r)^2)}\right]^{-1}, \tag{S24}$$

with $\mathcal{D} = 4(r-1)\tilde{\gamma}_{12}^2\left[16\tilde{g}_{12}^2 + (1+r)^2\right] + (1+r)^3\left[16\tilde{g}_{12}^2 + (4\tilde{\gamma}_1\tilde{\gamma}_2 + r(2+r))\right]$. Figure S6 shows that the conditions that increase concurrence also give rise to $g_{12}^{(2)}(0) < 1$. It shows a photon correlation map that resembles very much Figure 2(a) of the main text, as well and Figure S5 above. This indicates that

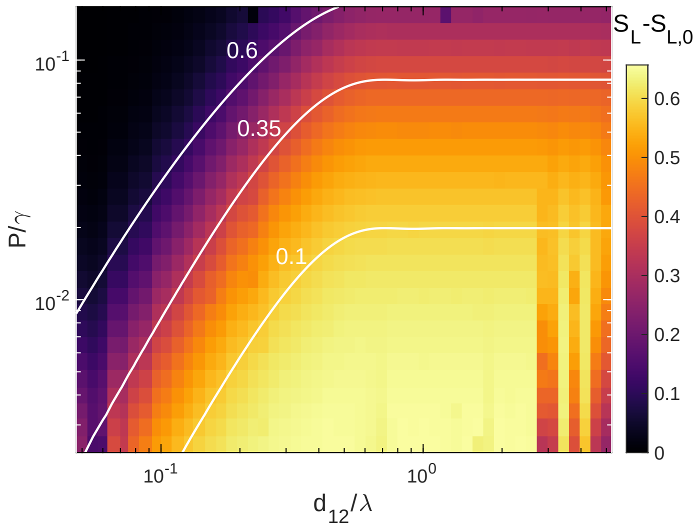

Fig. S5: Linear entropy enhancement, $S_L - S_{L,0}$, versus inter-emitter distance and pumping strength for the devices in Figure 2(a) of the main text.

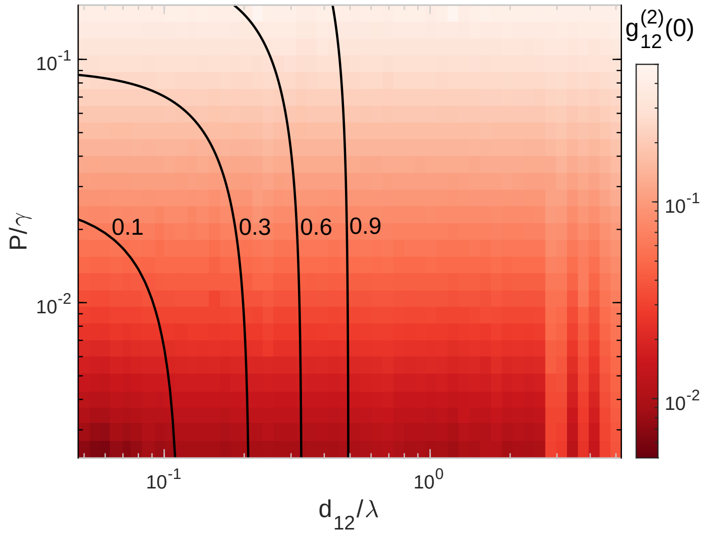

Fig. S6: Steady-state, normalized second-order cross correlation function, $g_{12}^{(2)}(0)$ versus d_{12} and P/γ for the ID devices in Figure 2(a). The colors render $g_{12}^{(2)}(0)$ in logarithmic scale from $5 \cdot 10^{-3}$ (dark red) to 1 (white). Isocurves of $g_{12}^{(2)}(0)$ in free-space are plotted in black solid lines.

antibunching (or subpoissonian statistics) takes place in the regions of large entanglement, and reduce the probability of simultaneous two-photon emission in the system.

5 Binarization and experimental realization

The designs obtained from our topology optimization algorithm take intermediate permittivity values between the vacuum permittivity $\epsilon = 1$, and the maximum permittivity, that results from the iterative procedure itself, but always satisfies $\epsilon_{\max} \leq 9$. No value has been discriminated as long as convergence was guaranteed. However, with the purpose of a possible experimental realization of our designs, in this section

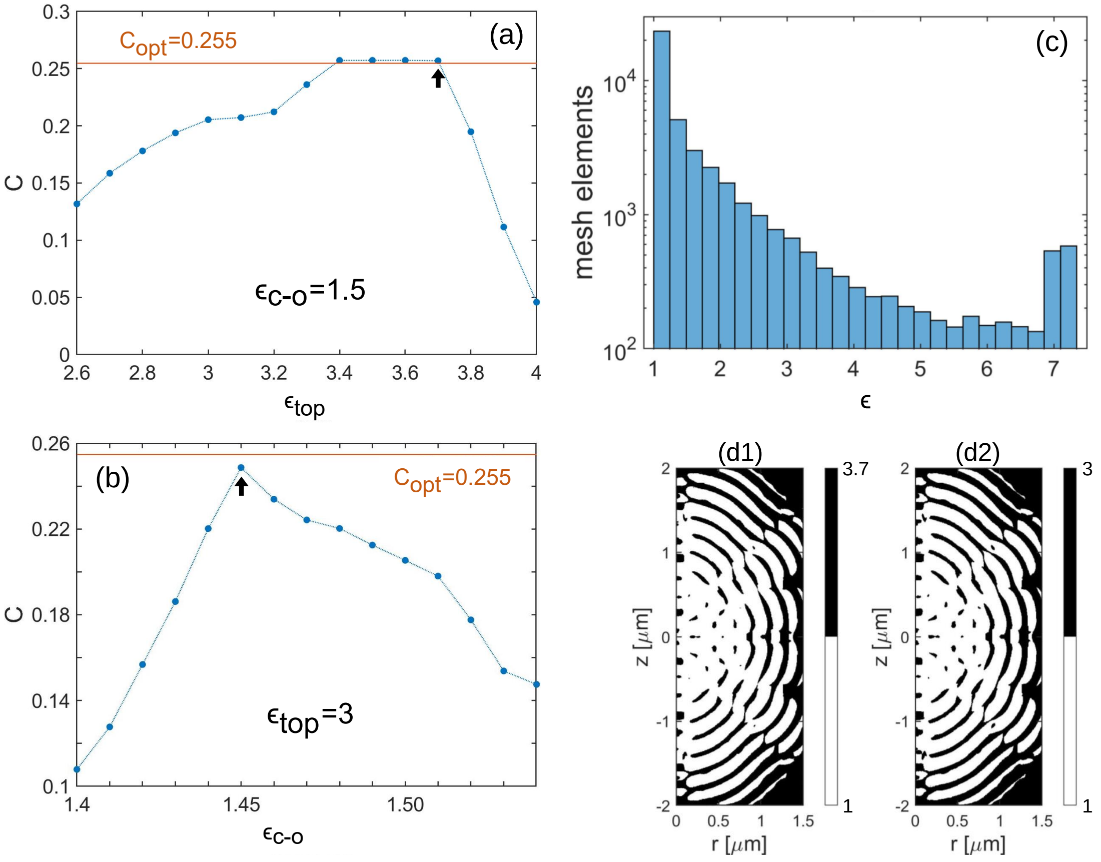

Fig. S7: Binarization of the ID cloak corresponding to point 4 in Figure 2(a) in the main text. (a) Concurrence versus top permittivity for $\epsilon_{\mathrm{c-o}} = 1.5$. (b) Concurrence versus cut-off permittivity for $\epsilon_{\mathrm{top}} = 3$. The horizontal continuous red lines in both panels indicate the concurrence value that results from the optimized dielectric structure. (c) Histogram that shows the number of mesh elements with a given permittivity value (with step $\delta\epsilon = 0.25$). (d) Binarized permittivity maps of the configurations indicated with the black arrows on panels (a) and (b): $\epsilon_{\mathrm{top}} = 3.7$, $\epsilon_{\mathrm{c-o}} = 1.5$ and $\epsilon_{\mathrm{top}} = 3$, $\epsilon_{\mathrm{c-o}} = 1.45$.

we introduce a binarization in the permittivity maps of the dielectric cloaks. The minimum permittivity is always set to 1 (air), while the material permittivity, ϵ_{top}, corresponds to the material that constitutes the cloak. In between, $\epsilon_{\mathrm{c-o}}$ sets the boundary between the two materials and marks the cut-off from which the rest of the values take any of the two previously chosen extremes. In this case, the binarized permittivity is given by

$$\epsilon_b(\mathbf{r}) = \begin{cases} 1 & \text{if } \epsilon(\mathbf{r}) \leq \epsilon_{\mathrm{c-o}} \\ \epsilon_{\mathrm{top}} = f\epsilon_{\mathrm{max}} & \text{if } \epsilon(\mathbf{r}) > \epsilon_{\mathrm{c-o}} \end{cases}, \tag{S25}$$

where $\epsilon(\mathbf{r})$ is the permittivity map that results from the optimization procedure.

Figure S7 displays the performance of our dielectric cloaks when the described binarization is applied. We selected the ID cloak that belongs to point 4 in Figure 2(a) of the main text ($d_{12} = 950\,\mathrm{nm}$, $P = 7\cdot 10^{-2}\gamma$) and represented the concurrence values obtained after implementing the binarization in the permittivity structure both in terms of ϵ_{top}, when the cut-off permittivity is fixed to $\epsilon_{\mathrm{c-o}} = 1.5$ (see panel (a)); and in terms of the cut-off permittivity $\epsilon_{\mathrm{c-o}}$, when the upper value is set to $\epsilon_{\mathrm{top}} = 3$ (see panel (b)). Note that the optimal concurrence is nearly achieved for several configurations of extreme and cut-off values.

The top permittivity value taken in the binarizations, ϵ_{top}, has been selected considerably far from the maximum resulting from the optimized case, $\epsilon_{\mathrm{max}} = 7.3$, this is, $f < 1$, due to the fact that most of the intermediate values are close to $\epsilon = 1$ in this design. This can be seen in the upper right histogram in panel (c) of Figure S7, where we represented the distribution of permittivity values within the ID cloak, this is, the number of mesh elements that have a permittivity value within a certain interval ($\delta\epsilon = 0.25$), covering

the entire range from air to ϵ_{max}. The distribution in panel (c), as well as results in panels (a) and (b), also reveal that, in spite of obtaining a design whose experimental realization entails drawbacks and difficulties, it is possible to convert it into a feasible structure without affecting its performance in the generation of entanglement. The binarized dielectric cloaks corresponding to maximum concurrence points indicated with black arrows in both panels (a) and (b) are also represented at the bottom right permittivity maps (d1) and (d2).

References

[1] E. del Valle, J. Opt. Soc. Am. B **28**, 228 (2011).
[2] L. Novotny and B. Hecht, *Principles of Nano-Optics*, 2nd ed. (Cambridge University Press, 2012).
[3] R. Bennett and S. Y. Buhmann, New Journal of Physics **22**, 093014 (2020).
[4] C. A. Downing, J. C. L. Carreño, A. I. Fernández-Domínguez, and E. del Valle, Phys. Rev. A **102**, 013723 (2020).
