# Supplementary figures and images for: Inverse-designed dielectric cloaks for entanglement generation
Source: Nanophotonics. 2022 Aug 22;11(19):4387–95. doi: 10.1515/nanoph-2022-0231 (PMC11501985; doi:10.1515/nanoph-2022-0231)

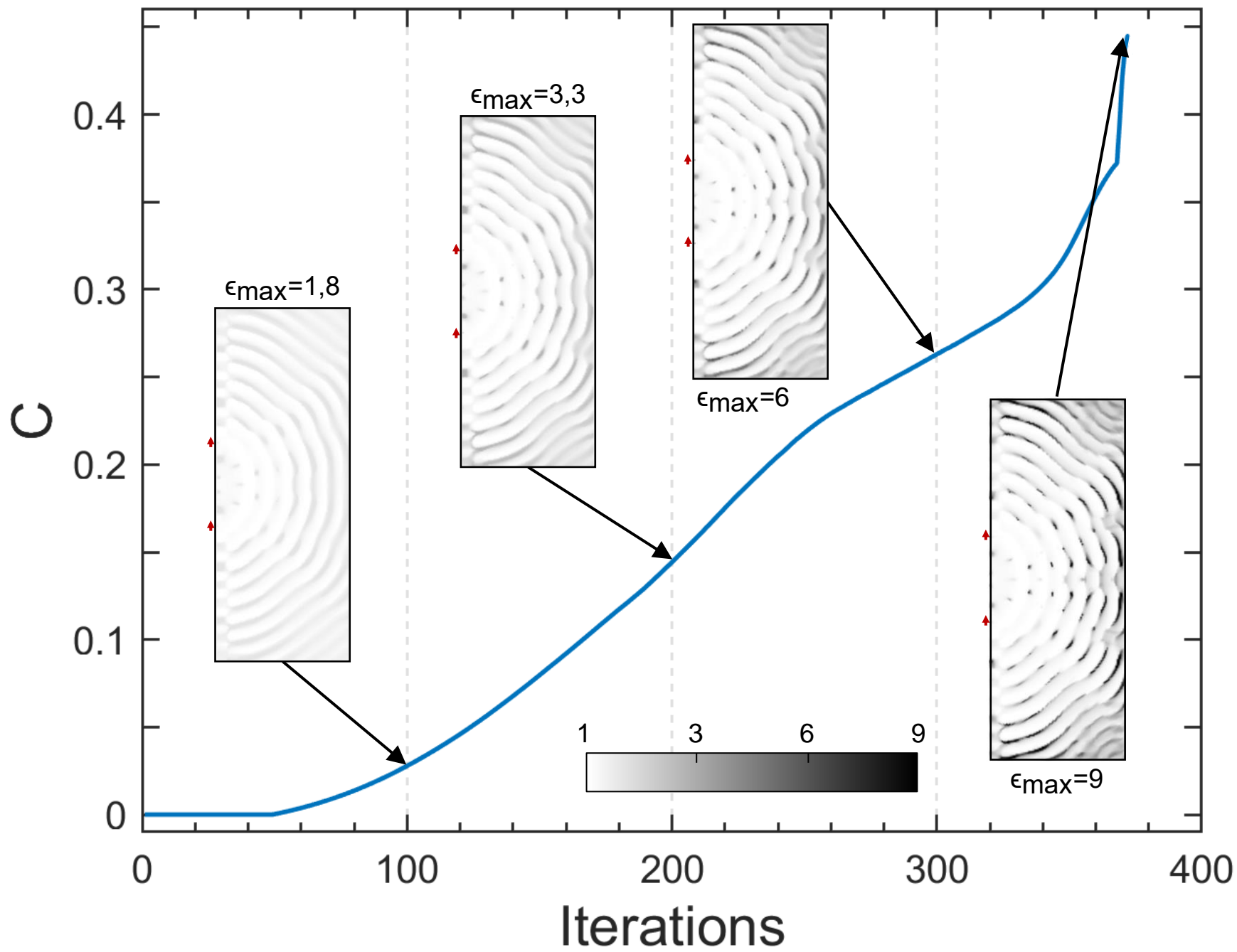

Supplement: Supplementary file 2 — Supplementary Material Details [file j_nanoph-2022-0231_suppl_002.zip › SM_invdesign_entanglement/FigS1.pdf]

2.

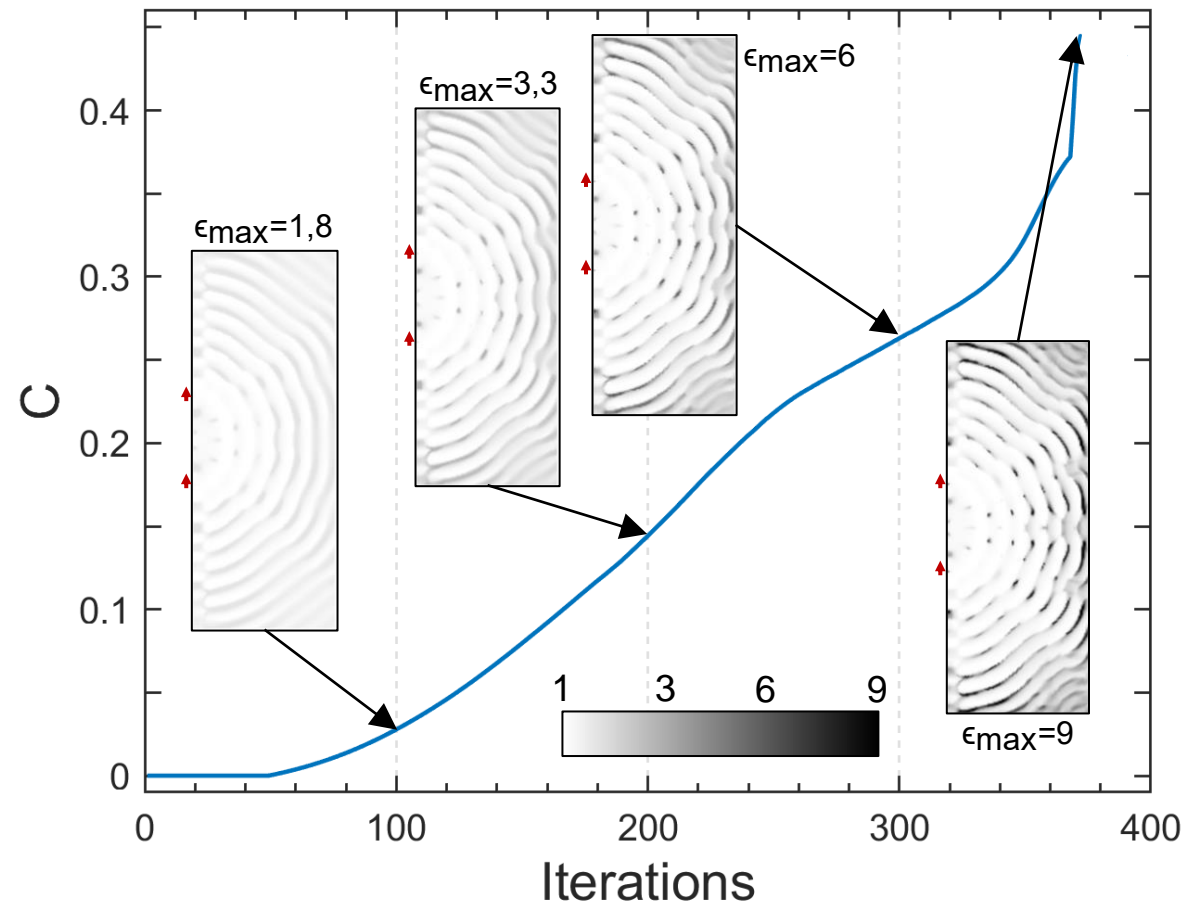

4.

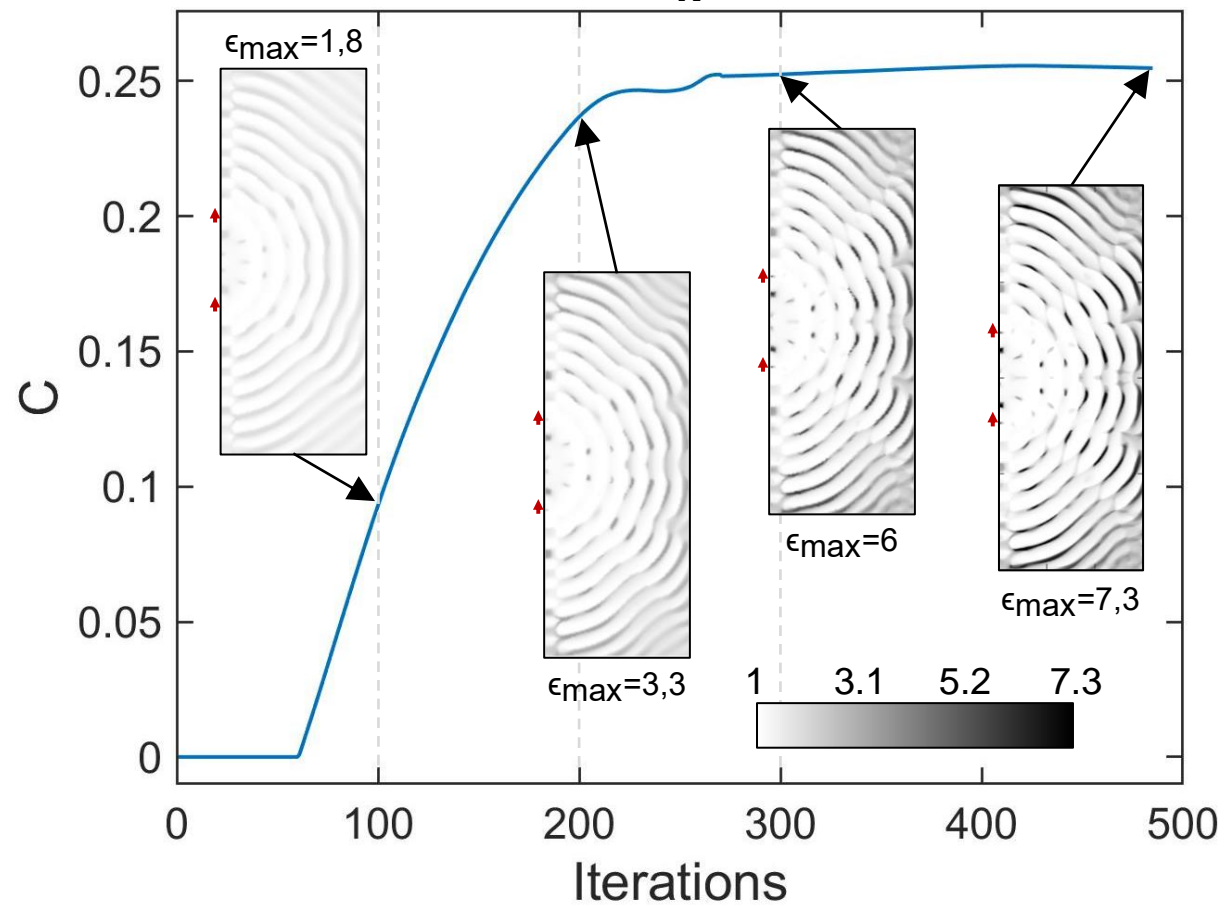

Supplement: Supplementary file 2 — Supplementary Material Details [file j_nanoph-2022-0231_suppl_002.zip › SM_invdesign_entanglement/FigS1a.pdf]

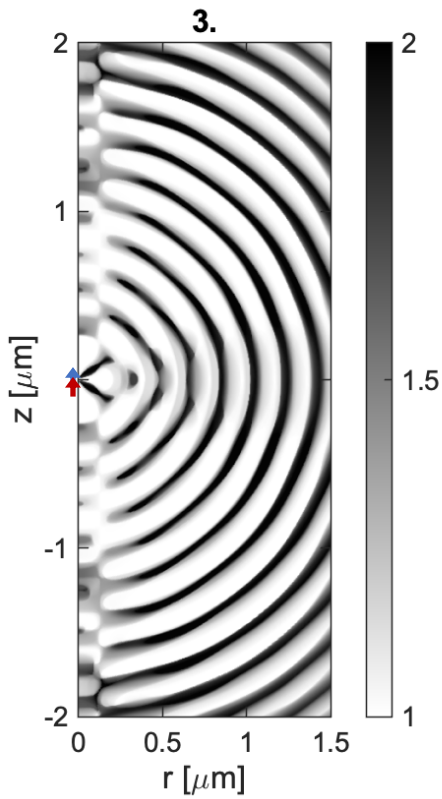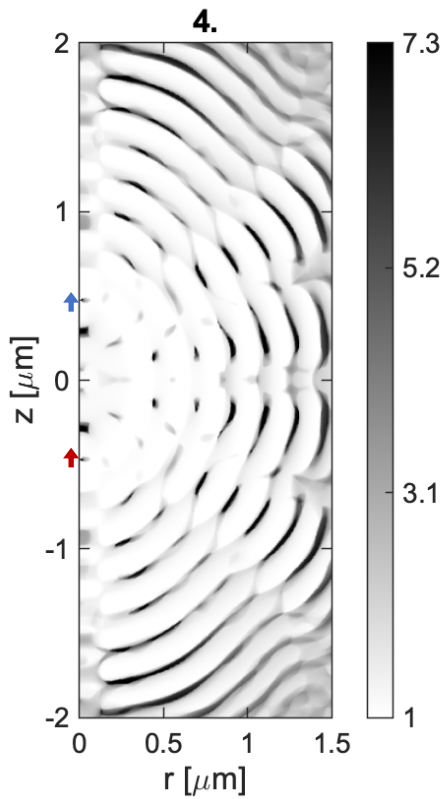

Supplement: Supplementary file 2 — Supplementary Material Details [file j_nanoph-2022-0231_suppl_002.zip › SM_invdesign_entanglement/FigS2.pdf]

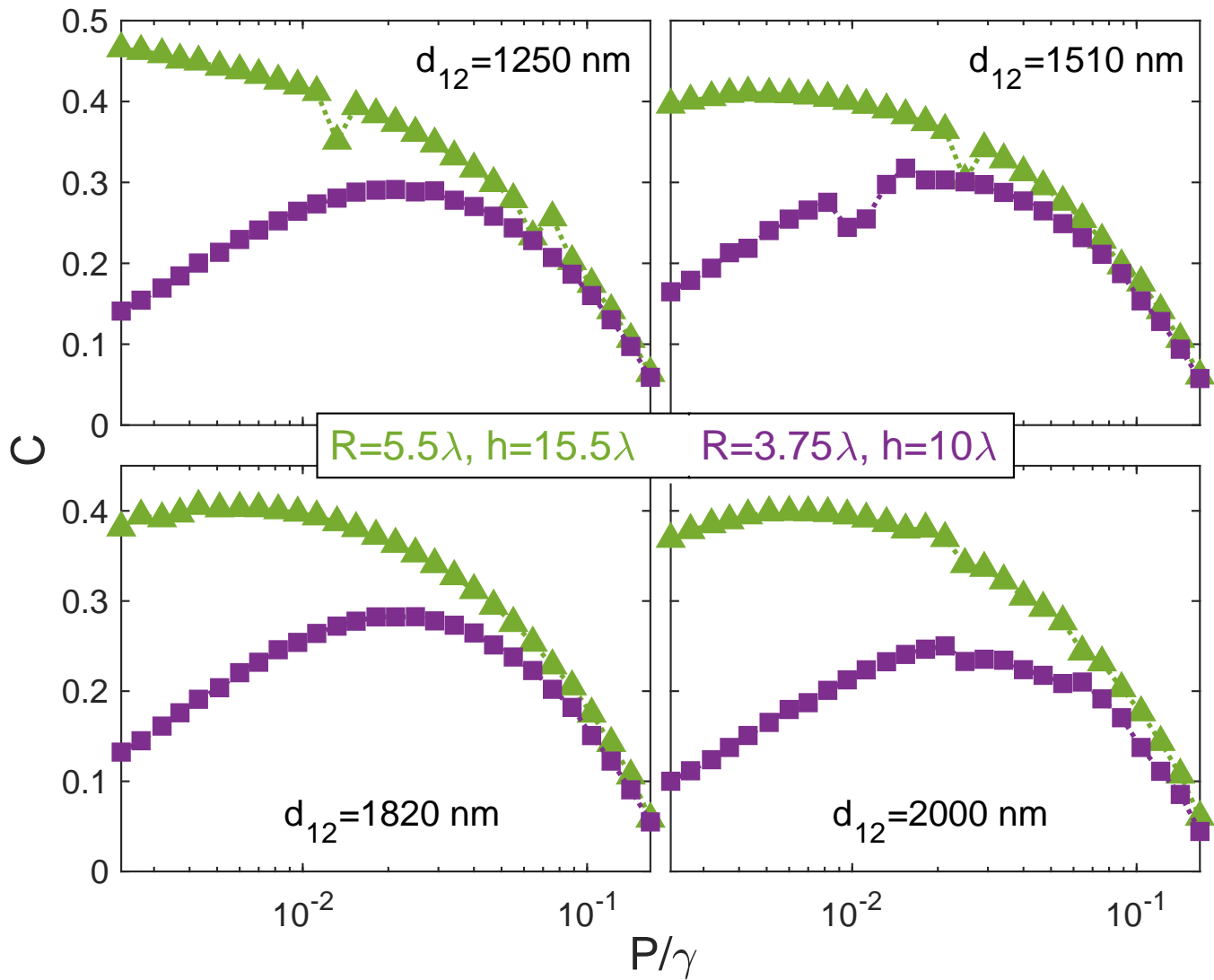

Supplement: Supplementary file 2 — Supplementary Material Details [file j_nanoph-2022-0231_suppl_002.zip › SM_invdesign_entanglement/FigS4.pdf]

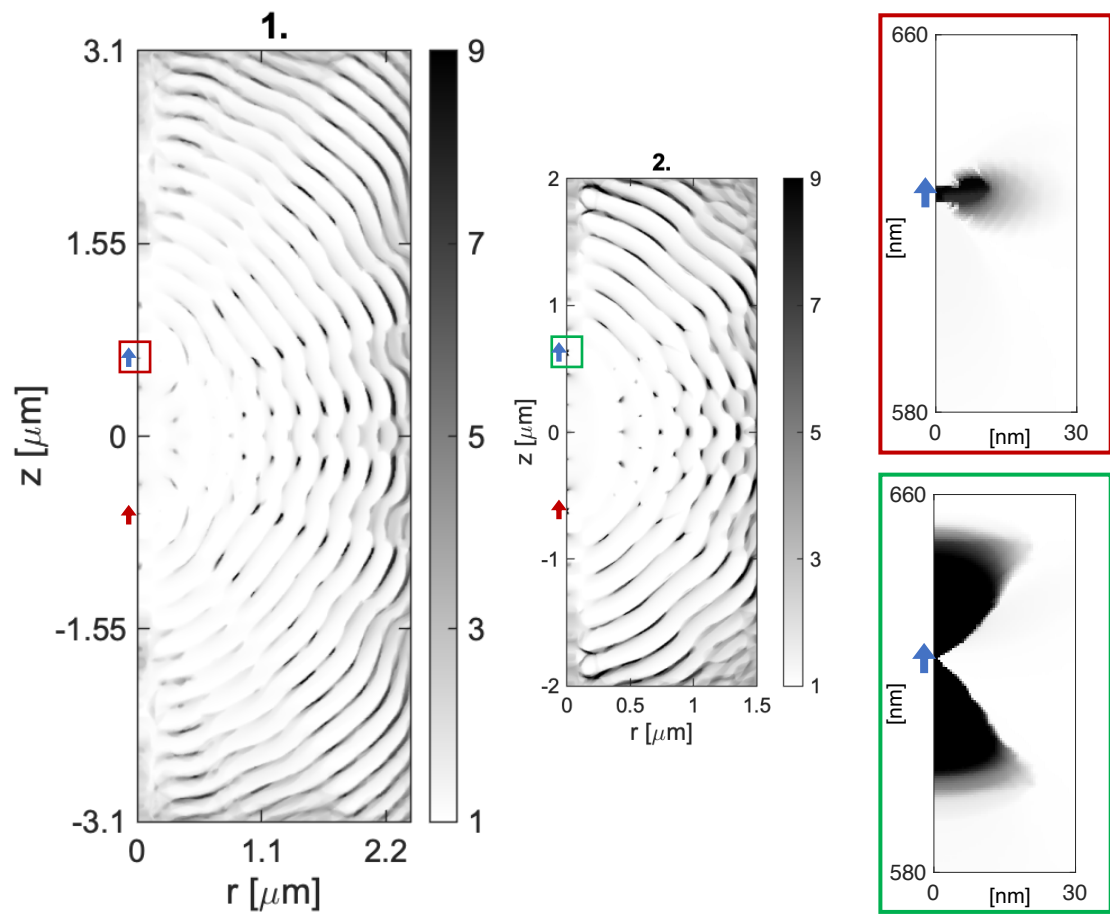

Supplement: Supplementary file 2 — Supplementary Material Details [file j_nanoph-2022-0231_suppl_002.zip › SM_invdesign_entanglement/FigS5.pdf]

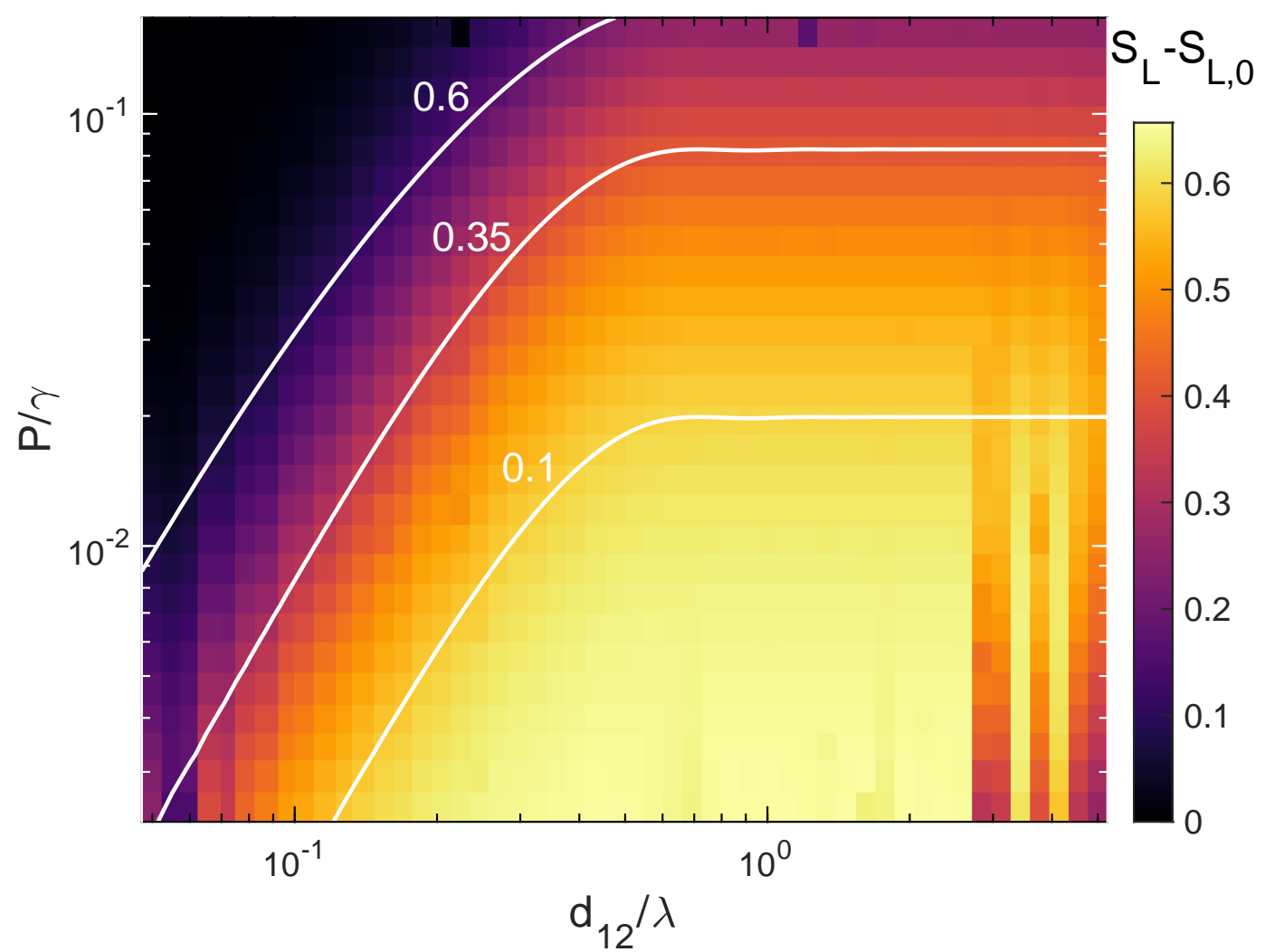

Supplement: Supplementary file 2 — Supplementary Material Details [file j_nanoph-2022-0231_suppl_002.zip › SM_invdesign_entanglement/FigS6.pdf]

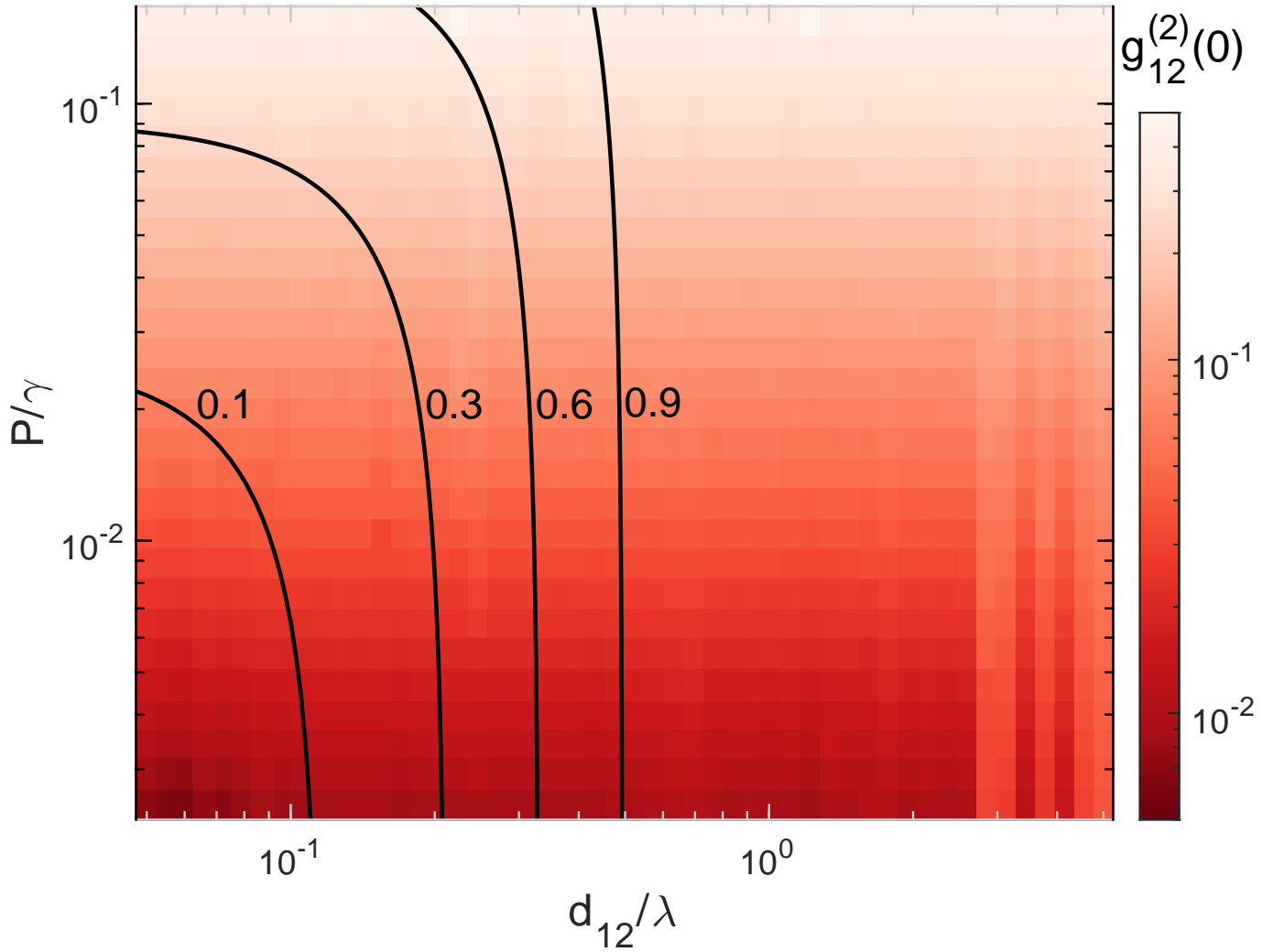

Supplement: Supplementary file 2 — Supplementary Material Details [file j_nanoph-2022-0231_suppl_002.zip › SM_invdesign_entanglement/FigS7.pdf]

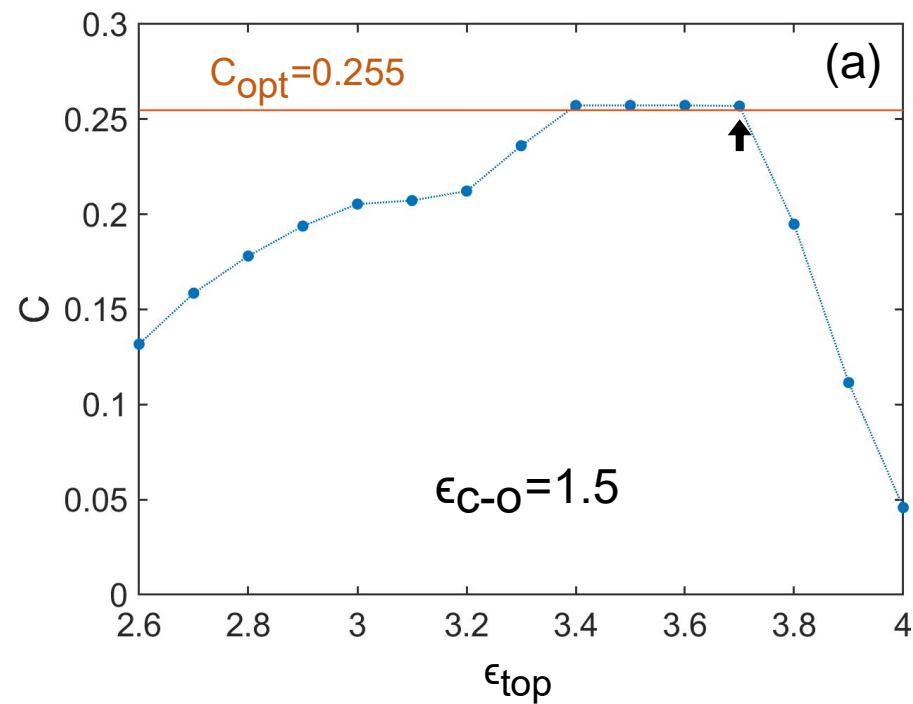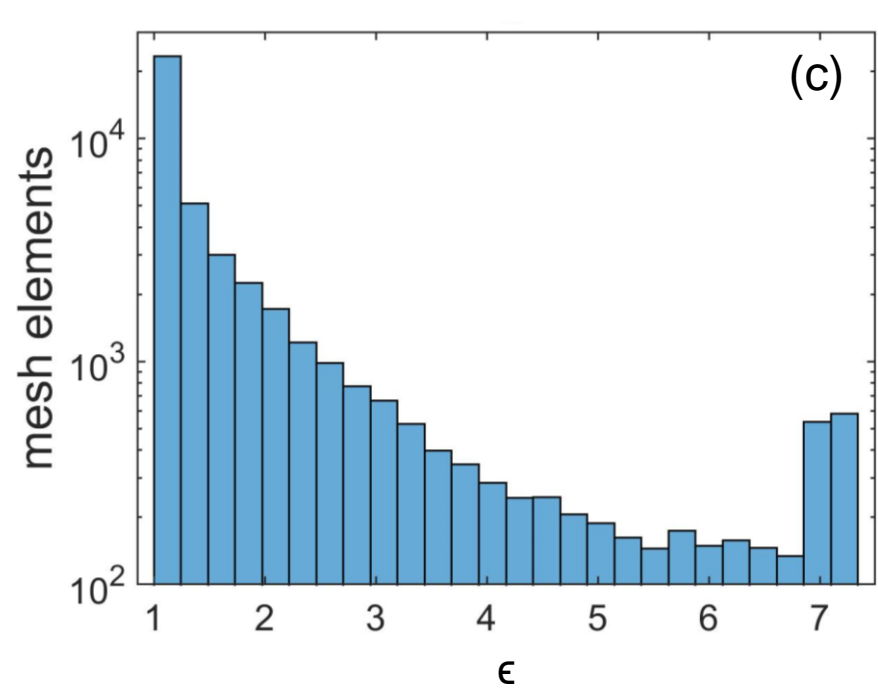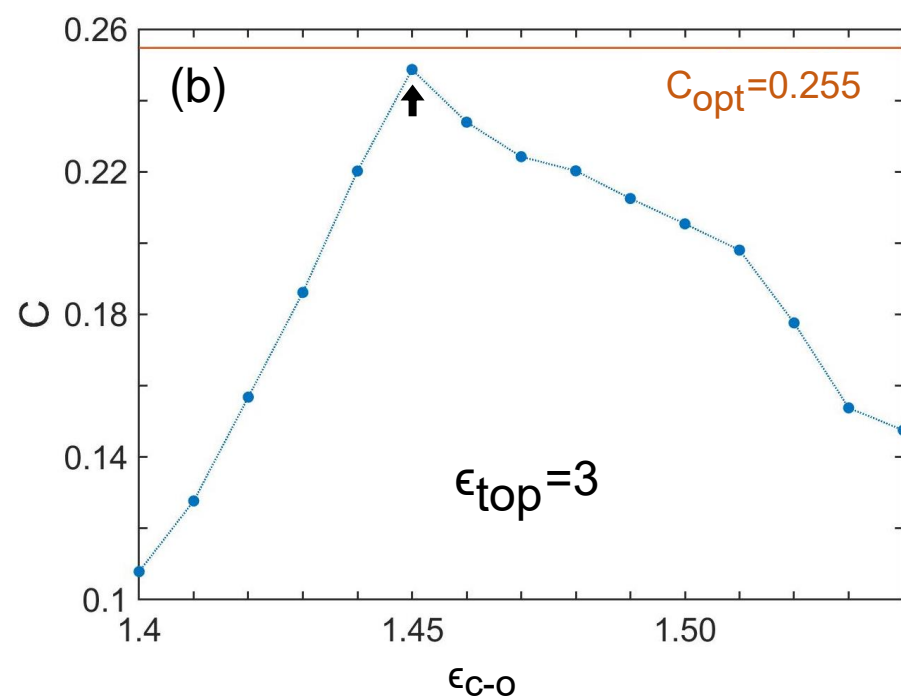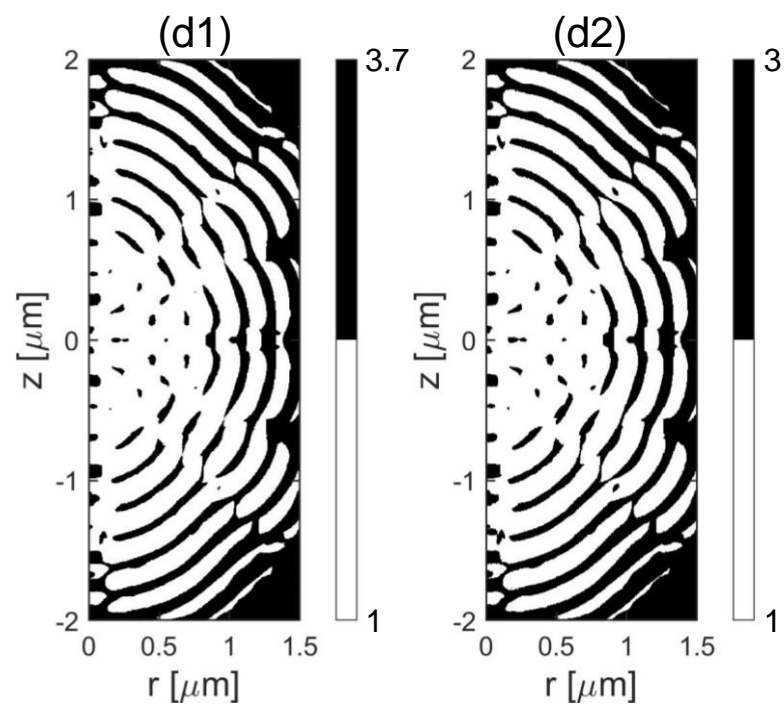

Supplement: Supplementary file 2 — Supplementary Material Details [file j_nanoph-2022-0231_suppl_002.zip › SM_invdesign_entanglement/FigS8.pdf]
